# Prediction of metabolic subphenotypes of type 2 diabetes via continuous glucose monitoring and machine learning

**DOI:** 10.1038/s41551-024-01311-6

**Published:** 2024-12-23

**Authors:** Ahmed A. Metwally, Dalia Perelman, Heyjun Park, Yue Wu, Alokkumar Jha, Seth Sharp, Alessandra Celli, Ekrem Ayhan, Fahim Abbasi, Anna L. Gloyn, Tracey McLaughlin, Michael P. Snyder

**Affiliations:** 1 Department of Genetics, Stanford University, Stanford, CA, USA.; 2 Systems and Biomedical Engineering Department, Cairo University, Giza, Egypt.; 3 Department of Medicine, Stanford University, Stanford, CA, USA.; 4 Department of Pediatrics, Stanford University, Stanford, CA, USA.; 5 Stanford Diabetes Research Centre, Stanford University, Stanford, CA, USA.; 6 Present address: Google LLC, Mountain View, CA, USA.; 7 Present address: Department of International Health, Johns Hopkins Bloomberg School of Public Health, Baltimore, MD, USA.; 8 These authors contributed equally: Tracey McLaughlin, Michael P. Snyder.

## Abstract

The classification of type 2 diabetes and prediabetes does not consider heterogeneity in the pathophysiology of glucose dysregulation. Here we show that prediabetes is characterized by metabolic heterogeneity, and that metabolic subphenotypes can be predicted by the shape of the glucose curve measured via a continuous glucose monitor (CGM) during standardized oral glucose-tolerance tests (OGTTs) performed in at-home settings. Gold-standard metabolic tests in 32 individuals with early glucose dysregulation revealed dominant or co-dominant subphenotypes (muscle or hepatic insulin-resistance phenotypes in 34% of the individuals, and β-cell-dysfunction or impaired-incretin-action phenotypes in 40% of them). Machine-learning models trained with glucose time series from OGTTs from the 32 individuals predicted the subphenotypes with areas under the curve (AUCs) of 95% for muscle insulin resistance, 89% for β-cell deficiency and 88% for impaired incretin action. With CGM-generated glucose curves obtained during at-home OGTTs, the models predicted the muscle-insulin-resistance and β-cell-deficiency subphenotypes of 29 individuals with AUCs of 88% and 84%, respectively. At-home identification of metabolic subphenotypes via a CGM may aid the risk stratification of individuals with early glucose dysregulation.

Type 2 diabetes (T2D) affects over 537 M adults globally^1^. Diabetes and prediabetes are defined by measures of glucose elevation, but the underlying physiology is complex and differs between individuals. It is traditionally suggested that individuals who develop T2D have both insulin resistance (IR) and β-cell dysfunction. However, we and others have shown that individuals with prediabetes and early T2D exhibit varying degrees of insulin resistance and β-cell dysfunction^2,3^, as well as defects in incretin action^4^ and hepatic glucose regulation^5^. These physiologic underpinnings of T2D are present before the onset of overt and prediabetes are defined by measures of glucose elevation, but the hyperglycaemia^6^, but they are not easy to identify outside of specialized metabolic research units. In particular, IR would be useful to detect in stages before overt hyperglycaemia because it is highly prevalent^7^ and increases risk not only for T2D but also for cardiovascular disease (CVD), stroke, hypertension, non-alcoholic liver disease and cancer^7–11^, and is largely reversible with lifestyle interventions such as weight loss and physical activity^12,13^.

We propose that classifying individuals with prediabetes, diabetes risk factors and newly diagnosed T2D according to their underlying metabolic physiology rather than level of glycaemia is possible and could enable a precision-medicine approach to diabetes prevention and treatment. Not only might metabolic subphenotypes pose differential risk for conversion to T2D, diabetes-related complications and/or CVD, they might determine relative efficacy of pharmacologic and lifestyle therapies. Multiple approved T2D treatments targeting specific physiologic components of glucose regulation now exist, virtually all of which have also been proven to prevent progression to T2D in high-risk individuals^14–17^. Efficacy in diabetes prevention varies between individuals, however, with more obese and younger individuals responding better to metformin over lifestyle interventions, and older less obese individuals responding better to lifestyle interventions^14^. Similarly, among non-insulin-treated diabetics starting monotherapy for T2D, females and patients with higher BMI responded better to thiazolidenediones, whereas males and patients with lower BMI responded better to sulfonylureas^18^. Heterogeneity in responses may reflect the heterogeneity in underlying physiology, since both pharmacologic and lifestyle interventions target different physiologic pathways to hyperglycaemia. Lifestyle interventions such as weight loss and exercise reduce insulin resistance^19,20^, whereas reducing dietary sugar and glycaemic load might benefit those with β-cell deficiency and/or incretin deficits; metformin reduces hepatic glucose production, acarbose prevents absorption of dietary carbohydrates in the small intestine, thiazolidenediones are powerful insulin sensitizers, and GLP-1 agonists augment β-cell insulin secretion and reverse insulin resistance via weight loss. Thus, phenotyping individuals according to underlying metabolic physiology might allow for targeted therapy at an early stage to prevent and/or treat T2D.

There is growing interest in identifying scalable cost-effective tests that capture the complexity of T2D phenotypes to inform precision-medicine approaches. These include partitioned genetic risk scores^21^, clinical and demographic features^22^, glucose metabolism biomarkers, clinical outcomes^23–28^ and even wearable devices^2,29^. The most common test for glycaemic disorders is the oral glucose-tolerance test (OGTT), which has been standardized and used globally for over 100 years. Limitations of fasting plasma glucose (FPG) in identifying early dysglycaemic states led to widespread use of the OGTT for epidemiological studies as well as clinical practice. Metrics such as the 2 h glucose, 1 h glucose, area under the curve and shape of the curve during a 3- or 5-timepoint OGTT have shown utility in predicting progression to T2D, development of microvascular disease, cardiovascular events and total mortality^30,31^. Furthermore, differences in the relationship between fasting and 2-h glucose elevations with hepatic IR, muscle IR and β-cell function highlighted the potential for OGTT to define underlying pathophysiology^30,32–37^. The burden of performing this test has limited its use clinically, particularly with regard to multi-timepoint analyses, which in 1980 were simplified to the fasting and 2-h timepoints alone. With the advent of continuous glucose monitor (CGM), it is possible to perform facile time-series measurements of glucose levels and OGTTs. We hypothesized that features of a 16-point glucose curve generated in response to standardized administration of an oral glucose load could identify the specific metabolic abnormalities underlying glucose dysregulation, which, if replicated by CGM-derived curves, would represent a scalable, practical and relatively cost-effective method that could facilitate individualized prevention and treatment of T2D, thereby enhancing precision approaches to diabetes.

In this work, we quantify the degree to which the physiologic basis for glucose dysregulation differs between individuals, who can be classified according to their metabolic subphenotypes. We also present a comprehensive framework using machine learning by which metabolic subphenotypes can be identified using features of the glucose time series extracted during a 16-point glucose-tolerance test. We then demonstrate that this can be performed at home using a CGM. The accuracy of this method, both in plasma and CGM, to identify underlying metabolic dysfunction is higher than standard measures of hyperglycaemia (for example, fasting glucose or HbA1c), existing biomarkers of metabolic disease (for example, HOMA) and genetic risk score. Together these results demonstrate the full potential of glucose measurements by CGM to define the pathophysiology underlying early dysglycaemic states.

## Results

### Overview of the study and cohorts studied

**Fig. 1 |:**
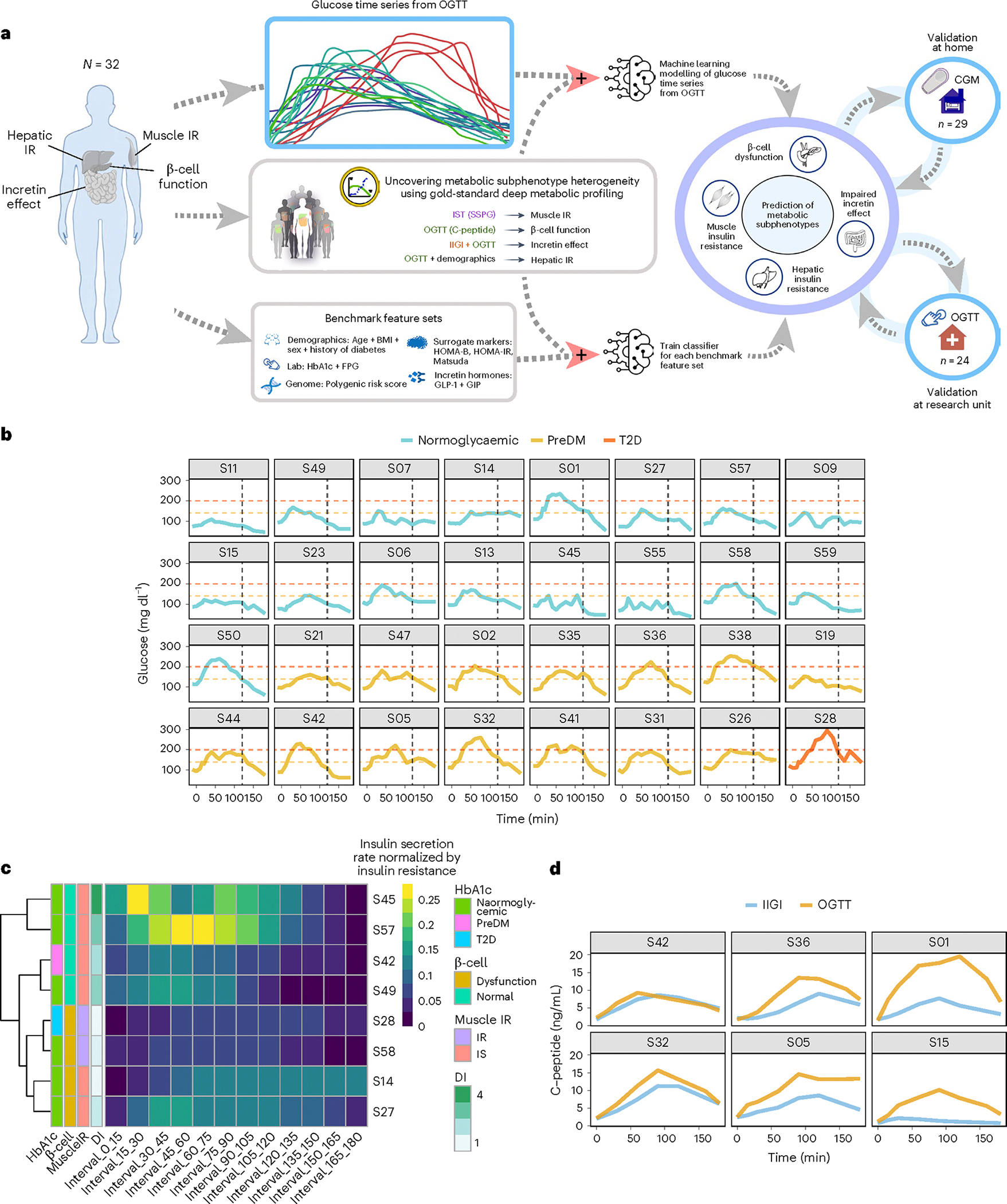
Metabolic subphenotyping study overview. **a**, Study design, where three cohorts were included in this study: (1) an initial cohort (*n* = 32) for training and testing the model, (2) a validation cohort (*n* = 24) and (3) an at-home CGM cohort (*n* = 29). Participants underwent deep metabolic profiling using gold-standard quantitative tests with the goal of assessing distinct physiologic phenotypes known to contribute to glucose dysregulation and T2D. Glucose time series following OGTT was used through a machine-learning (ML) approach to predict the four metabolic subphenotypes. **b**, Heterogeneity in glucose time series following a frequently sampled OGTT. All participants were normoglycaemic by fasting glucose <126 mg/dL but were categorized as normoglycaemic, prediabetes and T2D by HbA1c values. Time 0 on the *x* axis represents the time when participants consumed a 75 g glucola beverage. The black vertical dashed line represents the 120-min timepoint when glucose level is traditionally measured for clinical diagnosis of diabetes (glucose >200 mg/dL, red dashed line) or prediabetes (glucose 140–199 mg/dL, orange dashed line). **c**, Eight examples of insulin secretion rate (C-peptide deconvolution) normalized by insulin resistance (SSPG) used to infer β-cell function. **d**, Illustration of incretin effect variability. Six examples of C-peptide concentration during OGTT and IIGI. The incretin effect is calculated as the area between the C-peptide OGTT and IIGI divided by the area under C-peptide at OGTT. Left: poor incretin response (S42 and S32). Middle: moderate incretin response (S38 and S05). Right: robust incretin response (S01 and S15).

We enrolled 56 individuals without history of diabetes and fasting plasma glucose <126 mg dl^−1^, who were classified as having normoglycaemia (*n* = 33) or prediabetes (*n* = 21), as well as 2 with T2D according to American Diabetes Association HbA1c criteria (<5.7%, 5.7–6.4% and ≥6.5%, respectively). We then used a machine-learning approach to determine whether we could distinguish metabolic subphenotypes on the basis of the shape of glucose curves from a 16-point OGTT performed in the Stanford University School of Medicine’s clinical translational research unit (CTRU) (Fig. 1a) as well as the average of two OGTTs performed at home using a CGM (Fig. 5a). Metabolic physiology was characterized using gold-standard tests as described below.

Three cohorts were included in this study: (1) an initial cohort (*n* = 32) for training and testing the model, (2) a validation cohort (*n* = 24) and (3) an at-home CGM cohort (*n* = 29). For the main training cohort, 36 participants were enrolled and 32 completed all metabolic tests in the CTRU. These 32 participants were included in the final analyses as the initial training study cohort. An independent validation cohort of 24 participants was recruited separately and analysed. Finally, to test the feasibility of home CGM for metabolic subphenotyping, 29 participants (5 of the initial cohort and 24 of the validation cohort) participated in the at-home OGTT/CGM study and completed a minimum of two metabolic tests in the CTRU. This cohort also enabled us to compare the concordance of two home CGMs as well as home CGM versus CTRU CGM and CTRU plasma values during OGTT. The protocol was approved by the Stanford Internal Review Board and conducted according to the principles of the Declaration of Helsinki and Good Clinical Practice (Methods). The cohorts’ characteristics are summarized in Table 1 and Supplementary Table 1, and were well matched with an average age of 55 years, BMI of 26 kg m^−2^, relatively equal male/female sex ratio, 74% Caucasian and 27% Asian ethnicity, and HbA1c of 5.6%.

### Unique glycaemic responses to oral glucose load

To characterize the dynamic pattern of the glucose time series during the OGTT, we measured plasma glucose concentrations at 5–15-min intervals (16 timepoints) for 180 min following administration of a 75-g oral glucose load under highly standardized conditions (Methods) in the Stanford CTRU. We then performed deep metabolic profiling using gold-standard quantitative tests with the goal of assessing four distinct physiologic phenotypes known to contribute to glucose dysregulation and T2D: muscle IR, β-cell dysfunction, impaired incretin action and hepatic IR (Fig. 1a and Methods). We developed a machine-learning algorithm that used the glucose time series to predict metabolic subphenotypes (Fig. 1a).

As shown in Fig. 1b, OGTT glucose time-series profiles were very heterogeneous across individuals, despite similar classification based on OGTT 2-h plasma glucose levels (normoglycaemic: glucose < 140 mg dl^−1^, prediabetes: 140 ≤ glucose < 200 mg dl^−1^, diabetes: glucose ≥ 200 mg dl^−1^). The shape of the curve was remarkably different with regard to ascending and descending slopes, peak height and number of peaks, among other characteristics. As exemplified by participants S28, S42 and S01, some participants exhibited large rapid glucose spikes, whereas others (S21, S23) demonstrated little excursion at all. Some exhibited multiple glucose spikes (S42, S47, S55), whereas others had only a single spike (S32). Additional differences are apparent in Fig. 1b.

### Heterogeneous responses to metabolic testing

**Extended Data Fig. 1 |:**
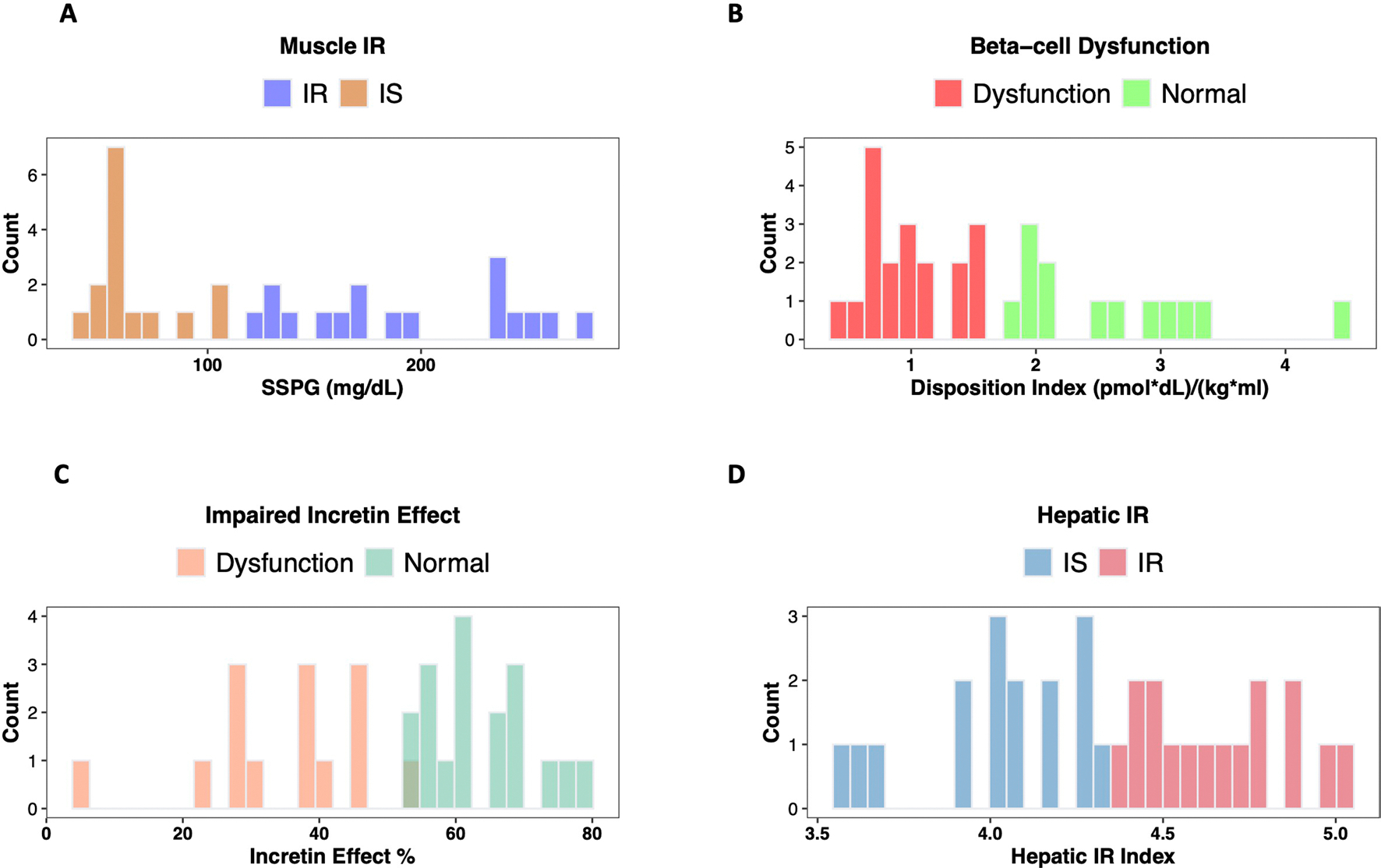
Distributions of classes of each metabolic subphenotype in the initial cohort. **(A)** muscle insulin resistance is categorized based on steady-state plasma glucose (SSPG), **(B)** β-cell dysfunction is categorized based on disposition index (DI), **(C)** impaired incretin effect is categorized based on the area under the C-peptide curves during OGTT, and IIGI, and **(D)** hepatic insulin resistance is categorized based on the hepatic insulin resistance index.

**Fig. 2 |:**
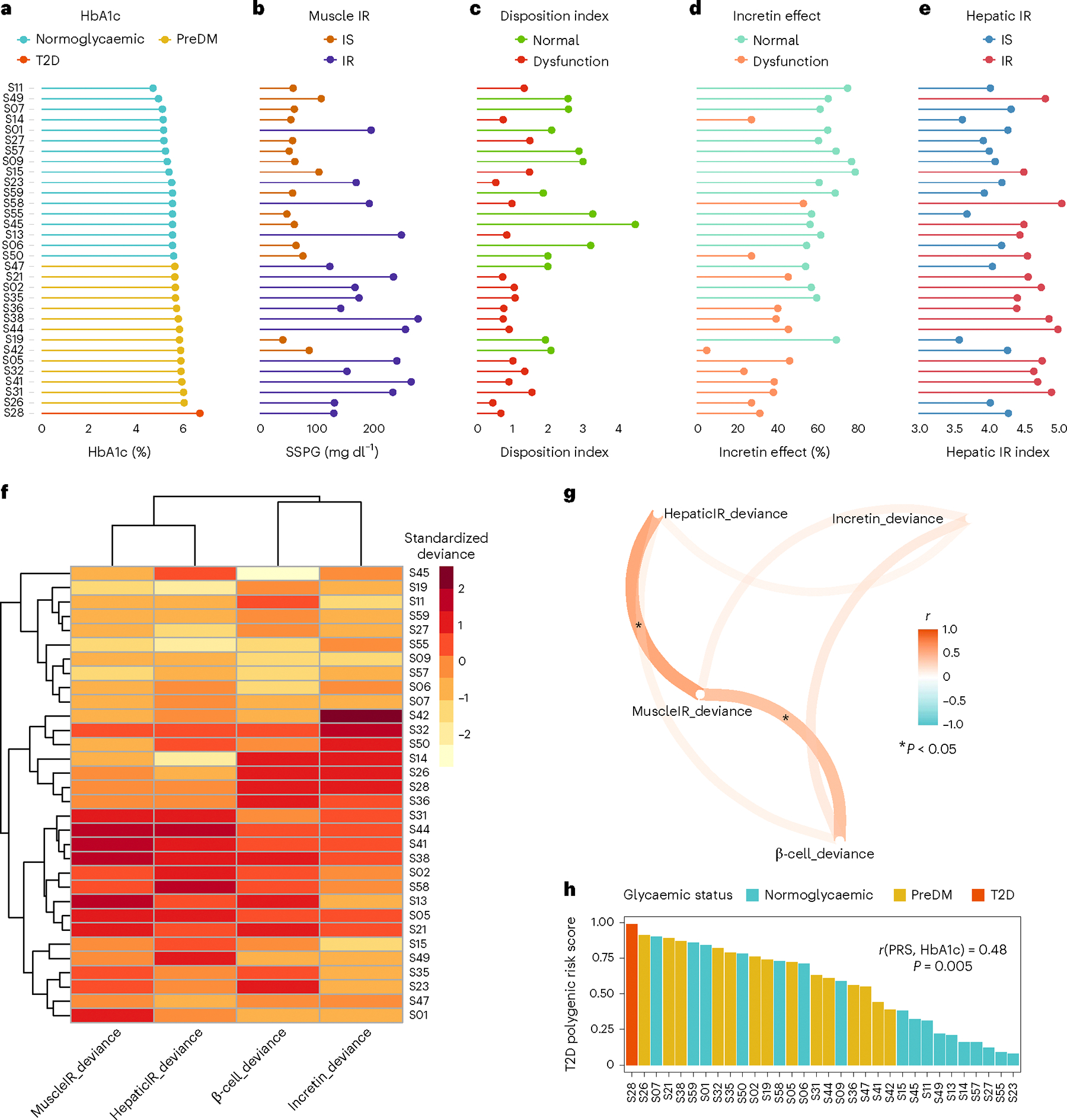
Heterogeneity in metabolism and determination of the dominant metabolic subphenotype. **a**, Detailed illustration of the four metabolic measures for our cohort sorted by the HbA1c. **b**, Muscle IR is categorized on the basis of SSPG (mg dl^−1^) values (IS when SSPG ≤ 120 and IR when SSPG > 120). **c,** β-cell function is categorized according to disposition index (DI) ((pmol dl)/(kg ml)): normal when DI > 2.2, intermediate when 1.2 ≤ DI ≤ 2.2, dysfunction when DI < 1.2. **d**, Incretin effect is categorized on the basis of the measured incretin effect (IE, %): normal when IE > 64, intermediate when 39 ≤ IE ≤ 64, and dysfunction when IE < 39. **e**, Hepatic IR is categorized on the basis of the hepatic-IR-index: IS when hepatic-IR-index > 3.95, intermediate when 3.95 ≤ hepatic-IR-index ≤ 4.8, and IR when hepatic-IR-index > 4.8. **f**, Heat map showing the standardized deviance score of each metabolic measure for each participant (Methods). β-cell function and incretin effect values have been reversed so that higher positive values represent a greater abnormality. High positive deviance (darker red) indicates greater deviance from study population average in the abnormal direction (for example, higher IR or lower incretin effect), while high negative deviance (lighter yellow) indicates a healthier metabolic phenotype than the average population. **g**, Pairwise correlation network showing the association between the standardized deviance of the four metabolic measures. Pearson correlation coefficient (−1<*r* < 1) was used to determine the strength of each relationship, where *r* = 1 ‘dark orange’ means strong positive correlation, and *r* = −1 ‘dark cyan’ means strong negative correlation. The asterisk symbol on edges means that the correlation is statistically significant (*P* < 0.05) between the two corresponding metabolic subphenotypes. **h**, T2D polygenic risk score (PRS) for all individuals in our cohort sorted in descending order and coloured by the glycaemic status measured using HbA1c. *P* values in **g** and **h** were calculated using *t*-test for Pearson correlations (evaluating the null hypothesis that the correlation is zero).

Muscle insulin resistance was measured by the modified insulin-suppression test (IST) and expressed as steady-state plasma glucose (SSPG)^38–40^. Participants were categorized as insulin sensitive (IS) if SSPG was <120 mg dl^−1^ and insulin resistant (IR) if their SSPG was ≥120 mg dl^−1^. Our designation of IR encompasses individuals above 50% of the distribution of SSPG among 490 healthy volunteers as previously described^41^. It includes those with ‘moderate’ elevations in SSPG who are more accurately defined as ‘non-IS’. This value also represented a natural cut-point in the distribution pattern of our study cohort (Extended Data Fig. 1a) and is clinically attractive as individuals with SSPG > 120 mg dl^−1^, even if moderately IR, respond to both lifestyle and pharmacologic treatments that target IR. As previously observed^41^, SSPG values spanned a wide range from 40 (participant S19) to 278 mg dl^−1^ (participant S38) (Fig. 2a and Supplementary Table 2). Importantly, in the current study, SSPG values indicating IR (>120 mg dl^−1^) or IS (<120 mg dl^−1^) varied despite glycaemic status. For example, some participants with elevated (prediabetes range) HbA1c had low SSPG values indicating insulin sensitivity, whereas others with normoglycaemia were quite insulin resistant (examples in Fig. 2a). These data emphasize that insulin resistance is not synonymous with glycaemic elevations.

**Extended Data Fig. 2 |:**
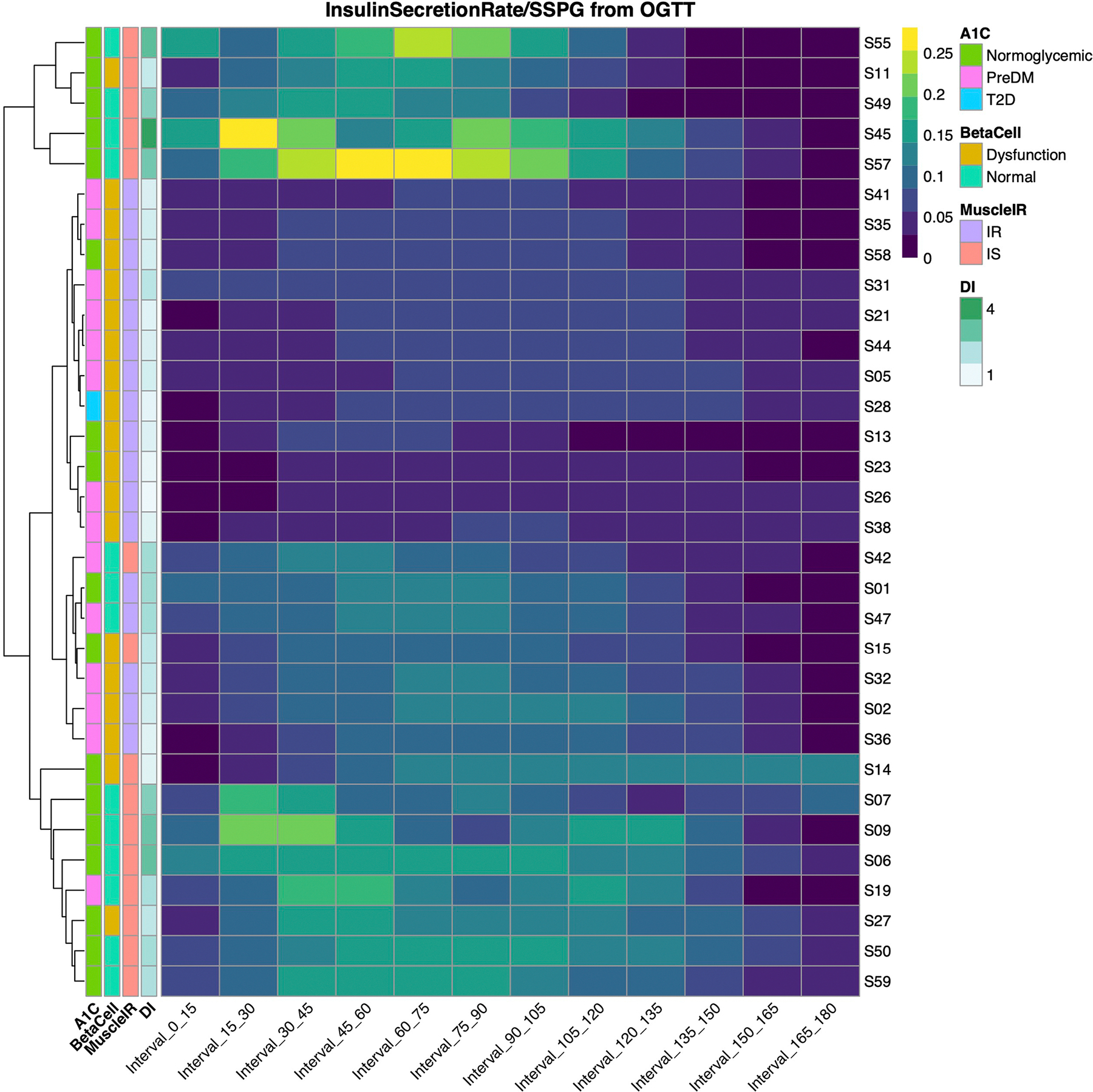
Insulin secretion rate measured during OGTT normalized by SSPG used to infer β-cell function. The insulin secretion rate was calculated using the C-peptide deconvolution method with C-peptide concentrations obtained at seven timepoints (0, 15, 30, 60, 90, 120, 180 min) during the OGTT, which was then adjusted for insulin resistance to generate a measure of β-cell function. Rows represent each participant, and columns represent time intervals in minutes relative to the start of OGTT. Yellow cells indicate time intervals with higher insulin secretion. Each participant is annotated with HbA1C class, β-cell function class (BetaCell), muscle insulin resistance class (MuscleIR), and disposition index (DI).

Insulin secretion rate was calculated using the widely used gold-standard C-peptide deconvolution method^42,43^, with C-peptide concentrations obtained at 7 timepoints (0, 15, 30, 60, 90, 120 and 180 min) during the OGTT. Insulin secretion was adjusted for insulin resistance (SSPG value) to generate a measure of β-cell function, referred to as disposition index (DI)^44,45^, also a validated gold-standard measure (Methods). In this work, DI < 1.58 (50th percentile in our cohort, Extended Data Fig. 1b) indicates dysfunctional β-cell function, whereas DI ≥ 1.58 indicates normal β-cell function. Figure 1c demonstrates the heterogeneity of β-cell function (DI) in eight participants at multiple time intervals during the OGTT (see Extended Data Fig. 2 for all participants, and Supplementary Table 2). Participants S45 and S57 had a high DI (4.47 and 2.88, respectively) at early time intervals following the 75-g oral glucose challenge, which demonstrates a ‘healthy’ β-cell function. On the other hand, participants S28 and S58 had extremely low DI (0.67 and 0.98, respectively) following the oral glucose load, which indicates dysfunctional β cells. As shown in Fig. 2a–c, β-cell function was generally higher among those with normoglycaemia. Among those with prediabetes, the β-cell function varied widely.

**Extended Data Fig. 3 |:**
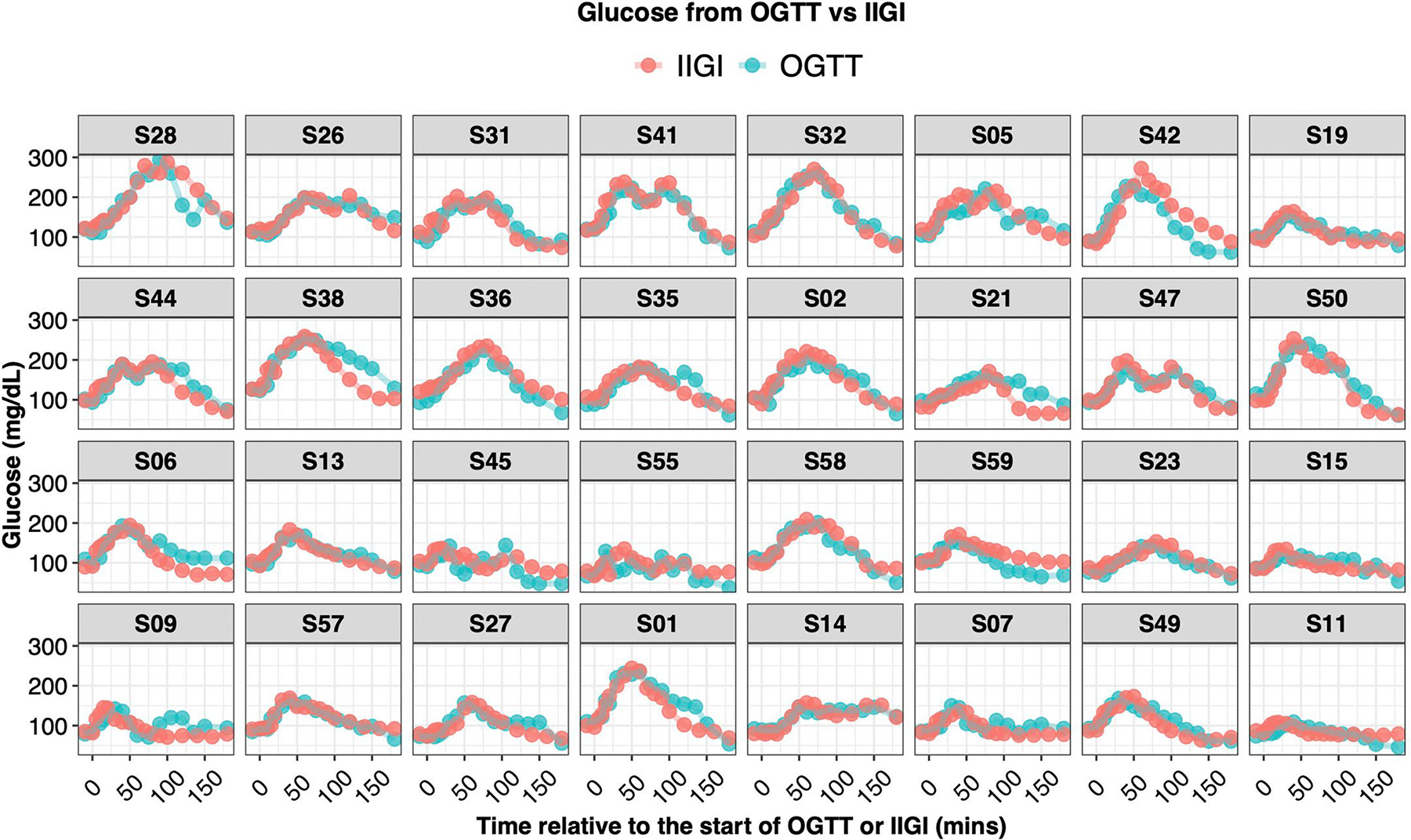
Concordance analysis of plasma glucose concentration during OGTT and IIGI. Glucose time-series from OGTT and IIGI are overlaid on each other, showing their high similarity. Each box represents a participant, the x-axis represents time in minutes relative to the start of OGTT or IIGI, and the y-axis represents glucose concentration in mg/dL. Average Pearson correlations between each individual’s glucose time-series from OGTT and IIGI equals 0.82, with a standard deviation equal 0.16.

**Extended Data Fig. 4 |:**
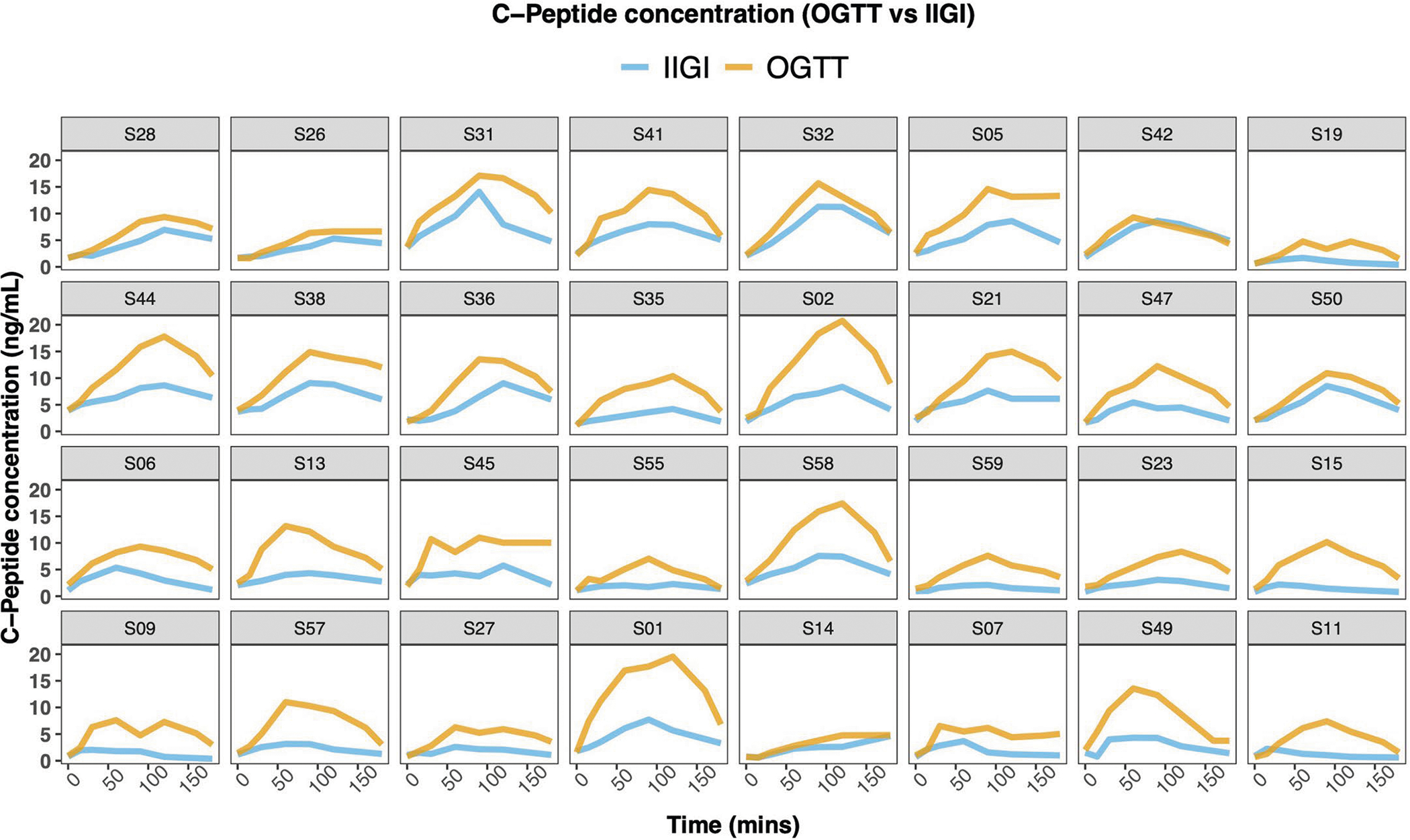
Plasma C-peptide concentration during OGTT and IIGI. C-peptide concentrations were obtained at seven timepoints (0, 15, 30, 60, 90, 120, and 180 min). Each box represents a participant, the x-axis represents time in minutes relative to the start of OGTT or IIGI, and the y-axis represents C-peptide concentration in ng/mL. The area between the two curves is calculated to infer the incretin effect.

**Extended Data Fig. 5 |:**
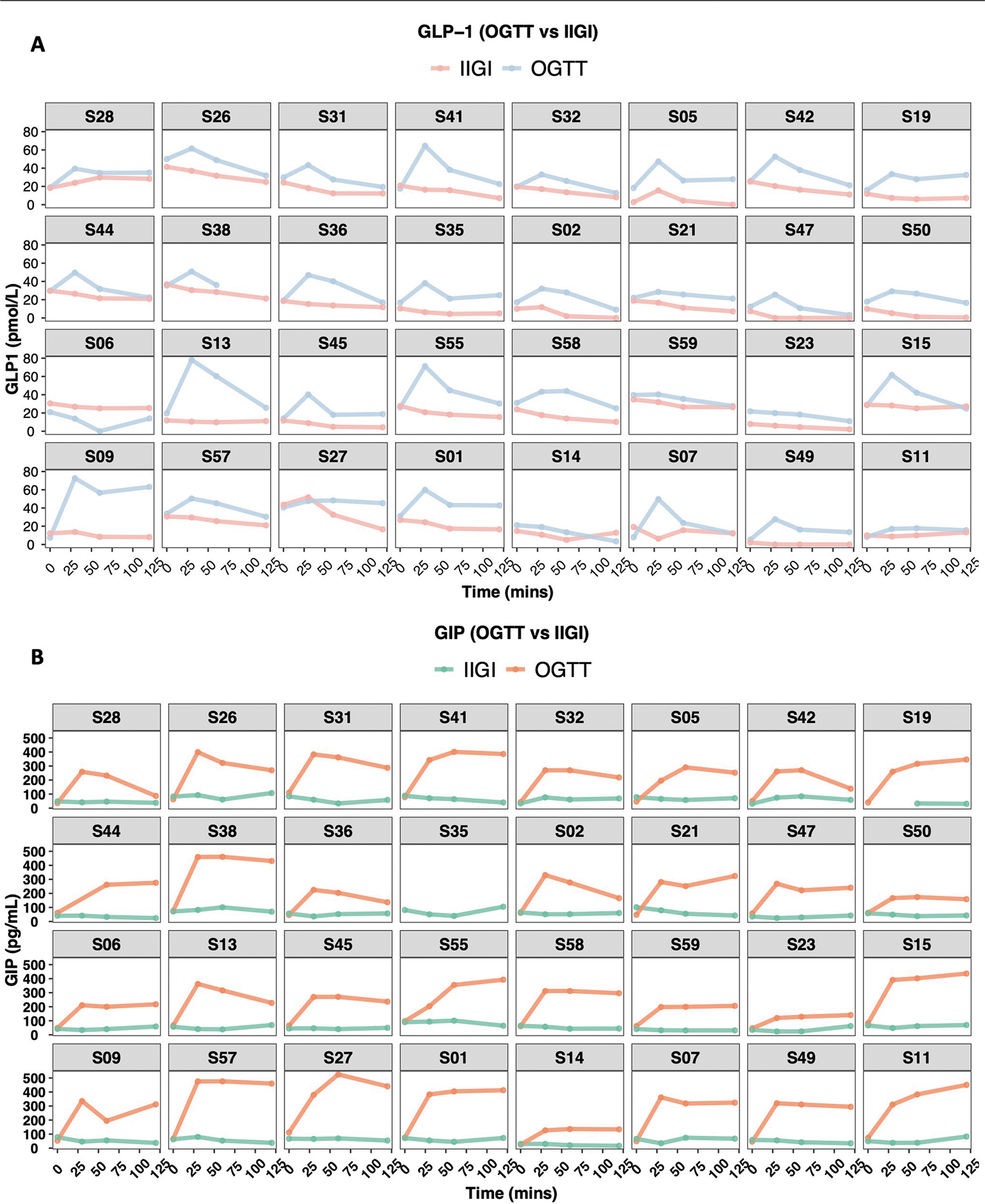
Total plasma Incretin hormones concentration during OGTT and IIGI. (**A**) GLP-1 concentration during OGTT and IIGI. **(B)** GIP concentration during OGTT and IIGI. The measurements were obtained at four timepoints (0, 30, 60, and 120 min). Participant S38 is missing the GLP-1 measurement at 120 min from OGTT. Participant S19 is missing GIP measurements at 0 and 30 min during IIGI. Participant S35 is missing GIP all measurements during IIGI. Each box represents a participant, the x-axis represents time in minutes relative to the start of OGTT or IIGI, and the y-axis represents GLP-1 or GIP concentration in pmol/L and pg/mL, respectively.

The incretin effect (IE) was measured using the gold-standard isoglycaemic intravenous glucose infusion test (IIGI)^46^ in which glucose is infused intravenously to mirror the glucose curve generated after oral glucose loading (see Extended Data Fig. 3 for concordance of glucose concentration between OGTT and IIGI). The difference in C-peptide concentrations during the OGTT relative to the IIGI was quantified at 7 timepoints (0, 15, 30, 60, 90, 120 and 180 min) to reflect the period of maximal hyperglycaemia (Extended Data Fig. 4). The degree to which C-peptide concentrations are elevated following oral versus intravenous glucose loading reflects the incretin effect (Methods and Supplementary Table 2). In this work, IE < 53.38% (50th percentile in our cohort; Extended Data Fig. 1c) indicates dysfunctional incretin effect, whereas IE ≥ 53.38 indicates normal incretin effect. The incretin response was extremely heterogeneous between participants and did not correlate with β-cell function or insulin resistance as shown in Fig. 2d,g. Figure 1d depicts the C-peptide concentrations during oral (OGTT) as compared to intravenous (IIGI) glucose loading in six participants. Participants S42 and S32 had little difference in C-peptide responses during the OGTT and IIGI tests, indicating a poor incretin effect (IE of 4.80% and 23.28%, respectively). In contrast, participants S01 and S15 demonstrated a robust increase in C-peptide during OGTT versus IIGI, consistent with a high incretin effect (IE of 64.92% and 78.59%, respectively). Notably, the secretion of incretin hormones, as measured by glucagon-like peptide-1 (GLP-1) and gastric inhibitory polypeptide (GIP) concentrations, also varied considerably between participants (Extended Data Fig. 5a,b). Interestingly, IE is significantly correlated with GIP at OGTT-2h GIP concentration (*r* = 0.53, *P* = 0.002), unlike GLP-1 which is not significantly correlated with IE (*r* = 0.3, *P* = 0.1).

Hepatic insulin resistance (hepatic IR) was calculated using the hepatic IR index, which is a formula validated against endogenous glucose production measured during euglycaemic–hyperinsulinemic clamp^47,48^ (Fig. 2e, Methods and Supplementary Table 2). In this work, a hepatic IR index < 4.35 (50th percentile in our cohort) indicates hepatic insulin sensitivity, whereas a hepatic IR index ≥ 4.35 indicates hepatic IR (Extended Data Fig. 1d). In our cohort, participants S58 and S44 had the highest hepatic IR with a hepatic IR index of 5.03 and 4.98, respectively. In contrast, participants S19 and S14 had the lowest hepatic IR with hepatic IR indices of 3.57 and 3.61, respectively.

### Dominant metabolic subphenotype calculation

For each participant, we attempted to determine which metabolic process was most impaired and thus classify individuals according to their ‘dominant’ or co-dominant metabolic subphenotype(s). This classification would be useful clinically to inform order of treatment with different therapeutics that target disparate physiologic processes. Classification entailed calculating the standard deviation values of each of the four metabolic measures as the deviation from the cohort mean (Methods). Positive deviations from the mean (shown in red or dark red on the heat map in Fig. 2f) indicate an unhealthy direction and negative deviations (shown as light orange or yellow on the heat map) indicate a healthy direction. Figure 2f highlights the differential deviance of each metabolic measure quantified per individual in our cohort. For example, for participant S42, the incretin effect was the metabolic subphenotype that was most deviant, and for participant S23, β-cell function was the most deviant—this method allows individuals to be classified according to the measure with the greatest deviance, which was termed their metabolic subphenotype. The majority of individuals (*n* = 16) had a single metabolic subphenotype that was most deviant (Methods and Supplementary Table 3), with β-cell function being the most frequent (*n* = 5), followed by muscle IR and incretin deficiency (*n* = 4 each), and then hepatic IR (*n* = 3). Thirteen participants had co-dominant metabolic subphenotypes (Supplementary Table 3), with hepatic IR and muscle IR, and β-cell and incretin deficiency having the highest prevalence (*n* = 4 each). Only three individuals (S35, S47, S57) could not be classified by this method as having a dominant or co-dominant metabolic phenotype. At the cohort level, we observed that individuals with high levels of muscle IR also had high levels of hepatic IR (*r* = 0.73, *P* = 10^−5^) (Fig. 2g). Indeed, a total of 13 individuals (34%) had either muscle, hepatic or combined IR. A total of 13 individuals had dominance or co-dominance in β-cell dysfunction or incretin deficiency (40%). β-cell dysfunction was significantly correlated with muscle IR (*r* = 0.6, *P* = 2 × 10^−4^) but not with impaired incretin effect (*r* = 0.33, *P* = 0.06) (Fig. 2g). Impaired incretin effect was not significantly associated with muscle or hepatic IR (*r* = 0.25, *P* = 0.17 and *r* = 0.21, *P* = 0.24, respectively). These data suggest that nearly all individuals in the normoglycaemic or prediabetic state can be classified according to underlying metabolic physiology with similar balance across IR subphenotypes and insulin-secretion subphenotypes.

### Polygenic risk score as a predictor of glycaemic level

Each participant was genotyped using a genome-wide array and the genetic predisposition for T2D was quantified by calculating the T2D polygenic risk score (PRS_T2D_) based on 338 independent signals for T2D risk, as previously developed and validated^49^ (Methods and Supplementary Table 4). Figure 2h shows that the PRS_T2D_ is associated with HbA1c level (*r* = 0.48, *P* = 0.005). A notable exception is female participant S07 (age at the time of the study is 37 years, BMI is 26.3 kg m^−2^) who had a high PRS_T2D_ of 0.9, despite normoglycaemic HbA1c (5.1%), muscle insulin sensitivity and β-cell function (SSPG = 60 mg dl^−1^, DI = 2.58; Fig. 2b,c), and intermediate incretin effect and hepatic IR (IE = 63.7%, hepatic IR index = 4.3; Fig. 2d,e). As expected, PRS_T2D_ was positively correlated, but did not reach significant levels, with SSPG (*r* = 0.26, *P* = 0.14), hepatic IR (*r* = 0.28, *P* = 0.1), HOMA-IR (*r* = 0.3, *P* = 0.08), BMI (*r* = 0.18, *P* = 0.29) and triglycerides (*r* = 0.22, *P* = 0.21). In addition, PRS_T2D_ was inversely correlated, but did not reach significant levels, with DI (*r* = −0.2, *P* = 0.25), IE (*r* = −0.16, *P* = 0.36), HOMA-B (a surrogate marker for β-cell function) (*r* = −0.32, *P* = 0.06) and high-density lipoprotein (HDL) (*r* = −0.28, *P* = 0.12).

### Features of the glucose response curve during OGTT identify metabolic subphenotypes

**Fig. 3 |:**
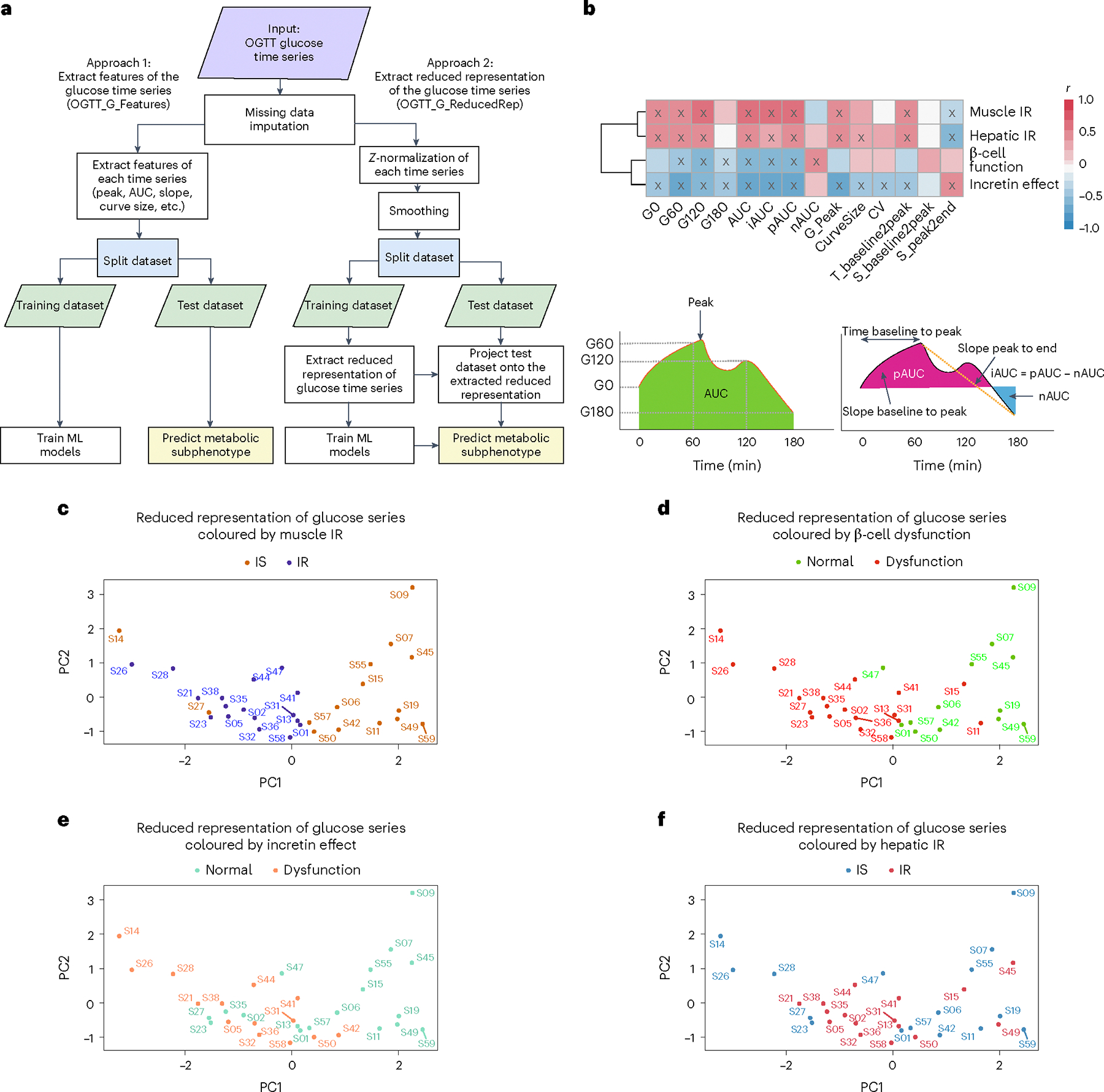
Features of the glucose time series identify metabolic subphenotypes. **a**, Machine-learning (ML) framework for predicting metabolic subphenotype using OGTT glucose time series. **b**, Top: relationship between OGTT glucose time-series features and metabolic subphenotypes. Pearson correlation coefficient (−1<*r* < 1) was used to determine the strength of each relationship, where *r* = 1 ‘dark red’ means strong positive correlation, and *r* = −1 ‘dark blue’ means strong negative correlation. The ‘X’ in some of the heat map cells means that the correlation between the curve feature and the corresponding metabolic subphenotype is statistically significant (*P* < 0.05). *P* values were calculated using *t*-test for Pearson correlations (evaluating the null hypothesis that the correlation is zero). Bottom: illustration of some of the glucose curve features. *G*_*t*_ denotes plasma glucose level at time *t*; CV, coefficient of variation; G_Peak, peak glucose level; CurveSize, length of the glucose time series; T_baseline2peak, time from baseline to peak value; S_baseline2peak, slope between baseline to the peak glucose leve; S_peak2end, slope between glucose values at the peak and at the end (at *t* = 180 min) (Methods). **c**–**f**, PCA of OGTT glucose time series, coloured according to classification using gold-standard metabolic tests for muscle IR (**c**) (measured by SSPG), β-cell function (**d**) (measured by the disposition index), incretin effect (**e**) and hepatic IR (**f**) (inferred by the hepatic IR index). Text on the plot represents participant ID.

Our main objective was to determine whether we could predict metabolic subphenotypes from the glucose time-series response obtained from the standardized OGTT test (Supplementary Table 5). To achieve this, we developed a robust machine-learning framework in which features were extracted from the OGTT glucose time series using two approaches: (1) engineered features from the OGTT glucose time series (OGTT_G_Features) and (2) a reduced representation method (OGTT_G_ReducedRep) that represents each normalized and smoothed OGTT curve with a vector of 2 dimensions (Fig. 3a). In the first approach (OGTT_G_Features), we extracted 14 features from the OGTT glucose time series (Methods). Figure 3b shows that multiple extracted features from OGTT glucose time series were significantly correlated with metabolic subphenotypes (*P* < 0.05, marked with an ‘X’ in Fig. 3b). Area under the curve (AUC) and incremental (i)AUC were the two highest significantly positively correlated features with muscle IR (*r* = 0.59 and *P* = 3 × 10^−4^, *r* = 0.57 and *P* = 6 × 10^−4^, respectively). Glucose levels at 120 min (G_120_) and iAUC were the top two negatively correlated with β-cell function (*r* = −0.59 and *P* = 4 × 10^−4^, *r* = −0.55 and *P* = 3 × 10^−3^, respectively). Positive (p)AUC and glucose peak levels (G_Peak) were the top two features significantly negatively correlated with the incretin effect (*r* = −0.72 and *P* = 6 × 10^−5^, *r* = −0.68 and *P* = 2 × 10^−5^, respectively). Finally, the slope from peak to the end at 180 min (S_peak2end) and baseline glucose levels (G_0_) were the top two parameters significantly correlated with hepatic IR (*r* = −0.51 and *P* = 0.002, *r* = 0.47 and *P* = 0.006, respectively).

**Extended Data Fig. 6 |:**
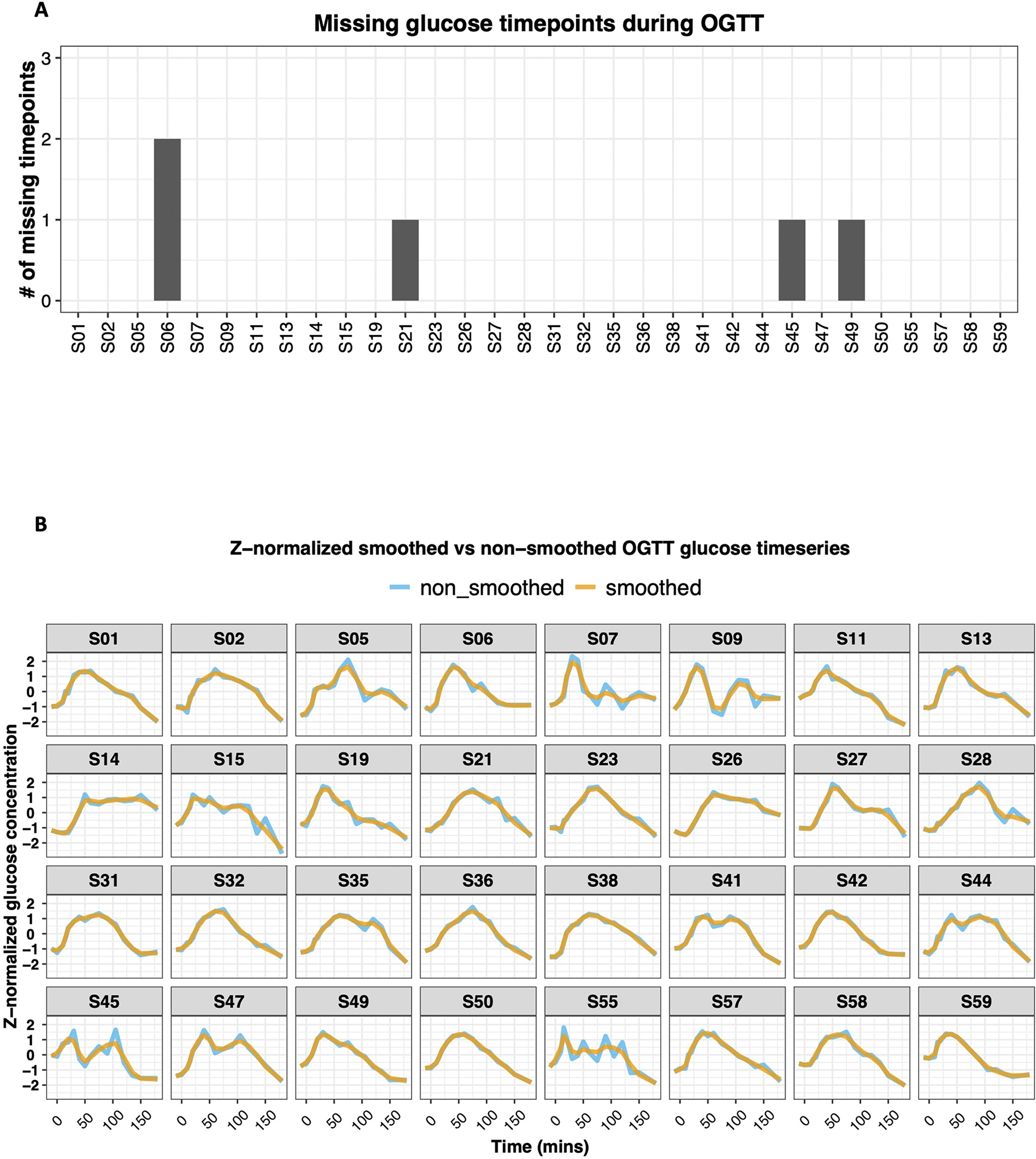
The effect of preprocessing steps employed on glucose time-series. **(A)** Shows the number of missing glucose timepoints for each participant. These timepoints were imputed using linear interpolation. As illustrated, 87.5% (28 out of 32 participants) of participants had complete time-series, 9.4% (3 out of 32 participants) were missing only one timepoint, and 3.1% (1 out of 32 participants) were missing 2 timepoints. **(B)** All smoothed glucose curves overlayed on top of the non-smoothed curve. As illustrated in the figure, smoothing has a minor effect on the curve in most cases. However, in cases where there are strong fluctuations (for example, S55), smoothing helps capture the general pattern rather than those immediate spikes and valleys.

In the second approach (OGTT_G_ReducedRep), we first *Z*-normalized and smoothed the OGTT glucose time series of 16 timepoints (Extended Data Fig. 6). We then extracted the reduced representation of the OGTT glucose time series (Methods). Interestingly, using the reduced representation of the OGTT glucose time series, there was a very clear separation of muscle IR and IS (Fig. 3c). In addition, the reduced representation of OGTT glucose time series readily distinguished the three classes of β-cell function (normal, intermediate and dysfunction) (Fig. 3d). Individuals with normal β-cell function were located on the positive side of the first principal component (PC1). In contrast, individuals with β-cell dysfunction were found on the negative side of PC1, and individuals with intermediate β-cell function were found in between. The same pattern was observed for incretin effect classes (Fig. 3e), where participants with normal versus impaired incretin effect were reasonably well separated. Participants with an intermediate incretin effect overlapped with the two more-extreme groups. On the other hand, hepatic IR classes were not separable using the reduced representation of the processed OGTT glucose time series (Fig. 3f).

**Extended Data Fig. 7 |:**
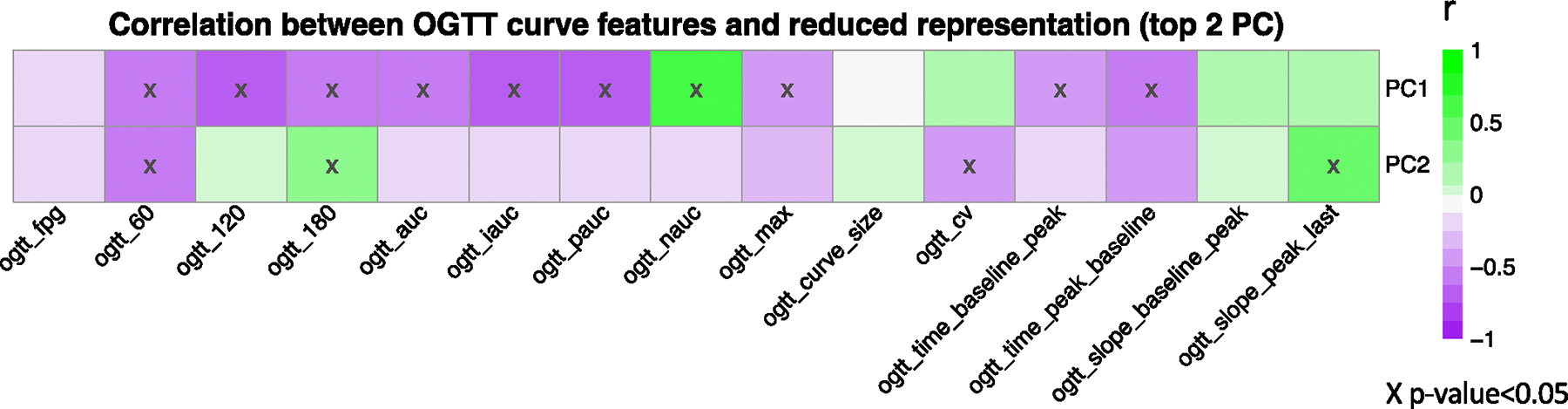
Relationship between the reduced representation of the glucose time-series and the extracted curve features. We calculated the correlations between the top 2 PCs (OGTT_G_ReducedRep) and the extracted features (OGTT_G_Features). Variances explained by PC1 and PC2 are 43.8% and 18%, respectively. As illustrated in the Figure, PC1 strongly negatively correlated with the incremental area under the curve (iAUC), and positively correlated with the negative area under the glucose curve (nAUC). This means, for example, that higher positive values on the PC1 are associated with lower iAUC.

We calculated the correlations between the top 2 PCs, OGTT_G_ReducedRep, and the extracted features, OGTT_G_Features (Extended Data Fig. 7). We found a relationship between the first two PCs and the 14 extracted curve features. The first two PCs are used to reduce the dimension in feature space while capturing the major variances (43.8% and 18% for PC1 and PC2, respectively). For example, higher positive values on PC1 are associated with lower iAUC, which from Fig. 3b, was associated with muscle IS. Figure 3c also confirms this relationship whereby muscle-IS participants are clustered towards higher positive values of PC1.

### Predicting metabolic subphenotypes from glucose time series using machine learning

**Extended Data Fig. 8 |:**
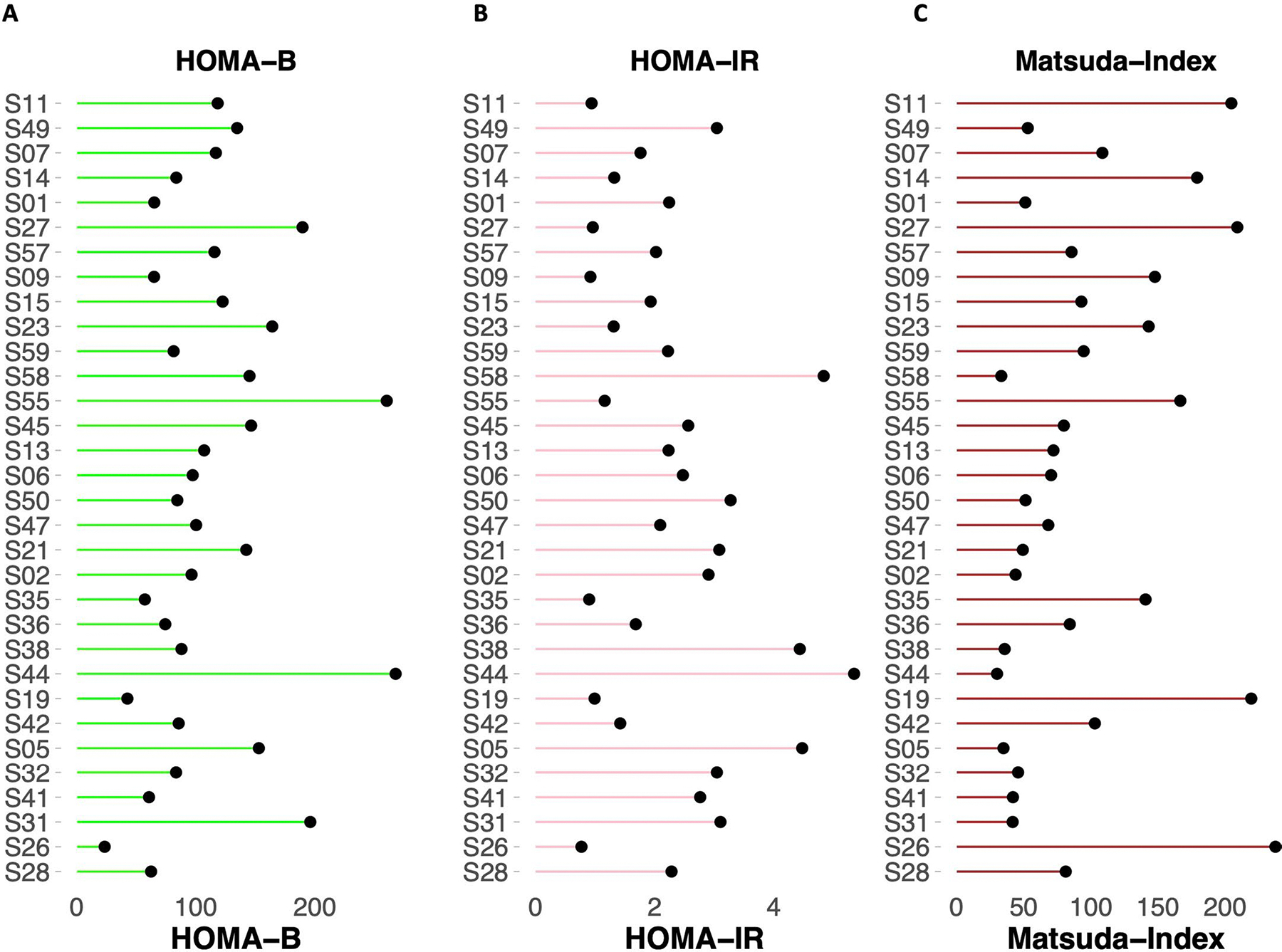
Common surrogate markers of insulin resistance and sensitivity measured on the initial cohort participants. **(A)** HOMA-B, **(B)** HOMA-IR, and **(C)** Matsuda Index.

The separability in the classes of muscle IR, β-cell function and incretin effect, using the reduced representation of the processed OGTT glucose time series suggested that a robust prediction of such classes could be achieved using a learning framework. For each feature extraction approach, OGTT_G_Features and OGTT_G_ReducedRep, the dataset was split into a training set and a test set. For each metabolic subphenotype, we optimized the hyperparameters of four different classifiers: random forest (RF), support vector machine with a radial basis function kernel (SVM-RBC), support vector machine with a linear kernel (SVM-linear) and logistic regression with L1 regularization (LR-L1). Models were evaluated comprehensively to ensure the generalizability and robustness of the trained models (Methods). We benchmarked the prediction of metabolic subphenotypes using the two sets of extracted features from the OGTT time series by comparing performance to surrogate metabolic measures in current use including (1) demographics (age, sex, BMI, ethnicity and participant family history for T2D); (2) T2D PRS and demographics; (3) lab (HbA1c and FPG) and demographics; (4) HOMA-B (Extended Data Fig. 8a), a surrogate marker for β-cell function, and demographics; (5) HOMA-IR (Extended Data Fig. 8b) and demographics; (6) Matsuda Index (Extended Data Fig. 8c), surrogate markers for muscle IR, and demographics; and (7) total GIP and GLP-1 concentrations at OGTT_2h (an optimized surrogate marker for incretin effect) and demographics. The performance of each model on each feature set and each metabolic subphenotype was evaluated using the area under the receiver operating characteristic curve (auROC), sensitivity, specificity, precision, F1-score and accuracy (Supplementary Table 6). For each metabolic subphenotype and for each feature set, models were ordered on the basis of F1-score and then sensitivity, and the top model was selected as the top performer. Table 2 summarizes all performance metrics of the top-performing model for each metabolic subphenotype and for each tested feature set. Statistical significance testing was performed between the measure of auROCs among all tested features, and OGTT_G_Features and OGTT_G_ReducedRep using the Wilcoxon rank-sum test (Supplementary Table 8). All *P* values were adjusted for multiple testing using the Bonferroni method.

**Fig. 4 |:**
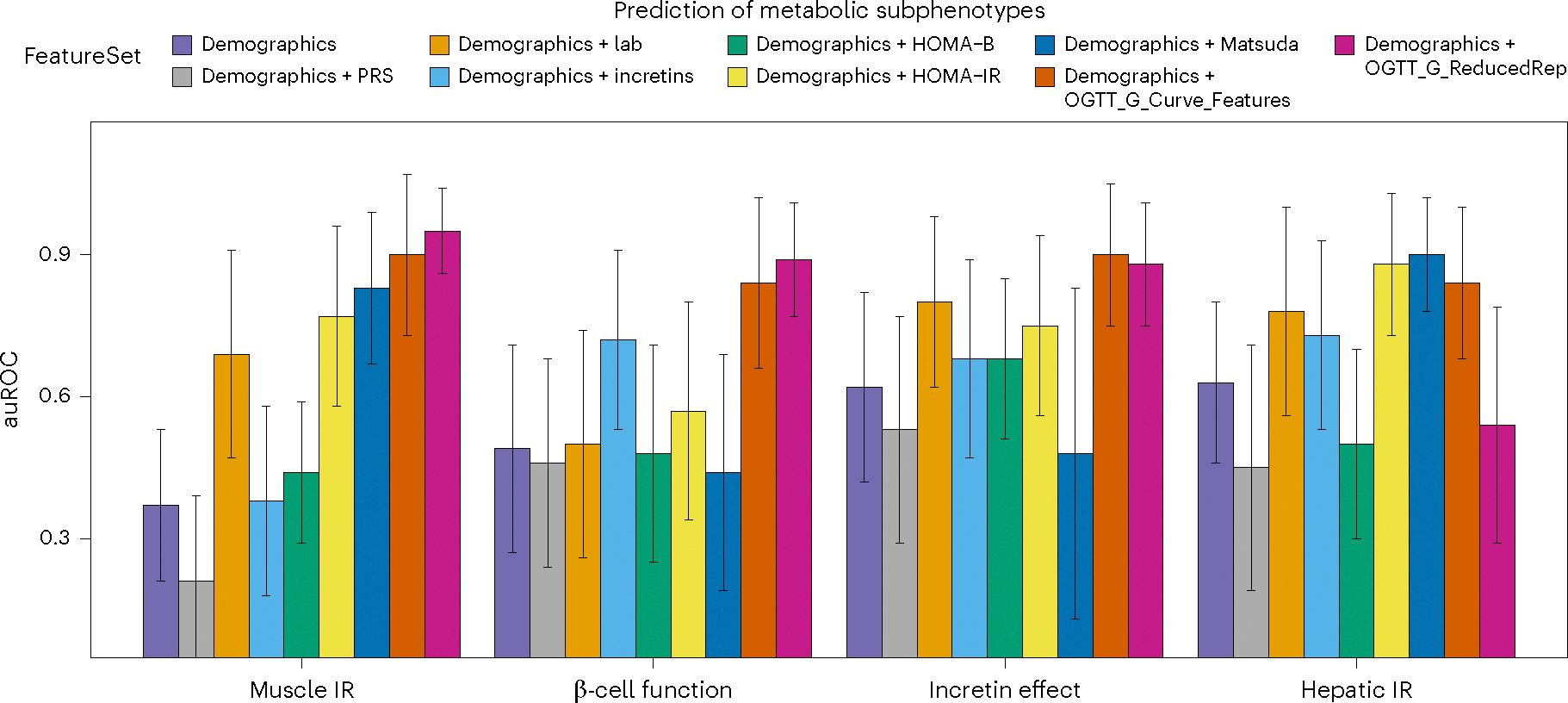
Benchmarking of metabolic subphenotyping prediction from features extracted from OGTT glucose time series versus existing surrogate markers. Bar graphs represent the average auROC of the best-performing model for each metabolic subphenotype and each corresponding feature set. Error bars represent the standard deviation of the measured auROC. In total, nine feature sets including demographics were evaluated for each metabolic subphenotype: two sets of features obtained from OGTT glucose curve (OGTT_G_Features and OGTT_G_ReducedRep), T2D polygenic risk score (PRS), and six measures in current use including demographics alone (age, sex, BMI, ethnicity and participant family history for T2D), lab (HbA1C and FPG), HOMA-B, HOMA-IR and Matsuda Index (both are surrogate markers for insulin resistance), and incretins (total GIP and GLP-1 concentrations at OGTT_2h, which are optimized surrogate markers for incretin effect). Four classifiers were trained on the training set and the *y* axis represents the auROC of the best-performing classifier on the test set for each metabolic subphenotype and each feature set. Statistical significance of differences between the measure of auROCs among all tested features and OGTT_G_ReducedRep was determined using the Wilcoxon rank-sum test.

Figure 4 summarizes the auROC of the best-performing classifier for metabolic subphenotypes using each feature set. Muscle IR was predicted with an extraordinarily high auROC of 0.95 using OGTT_G_ReducedRep, while OGTT_G_Feature predicted muscle IR with an auROC of 0.90. Importantly, OGTT_G_ReducedRep had significantly higher predictive power than any of the currently used features to predict muscle IR, including our optimized measure of demographics + Lab (auROC = 0.69, *P* = 1.2 × 10^−19^), demographics + HOMA-IR (auROC = 0.77, *P* = 7 × 10^−15^) and demographics + Matsuda Index (auROC = 0.83, *P* = 2.9 × 10^−8^). β-cell dysfunction was predicted from OGTT_G_ReducedRep with an auROC of 0.89, which is significantly higher than that of demographics + Lab (auROC = 0.5, *P* = 7.6 × 10^−24^), demographics + HOMA-B (auROC = 0.48, *P* = 1.28 × 10^−25^) and demographics + HOMA-IR (auROC = 0.57, *P* = 7.8 × 10^−20^). Incretin deficiency could be predicted using OGTT_G_ReducedRep with an auROC of 0.88 and using OGTT_G_Features with an auROC of 0.90, which were higher than those of demographics + Incretins (auROC = 0.68, *P* = 2.6 × 10^−12^) and demographics + Lab (auROC = 0.8, *P* = 2.9 × 10^−3^). For hepatic IR, OGTT_G_Features showed a promising performance (auROC = 0.84); however, Matsuda Index (auROC = 0.90, *P* = 0.86) and HOMA-IR (auROC = 0.88, *P* = 1) showed superior performance but not statistical significance. Thus, models for different metabolic subphenotypes could be built from features of the OGTT glucose time series.

### Validation of metabolic subphenotype predictions in independent cohort

**Fig. 5 |:**
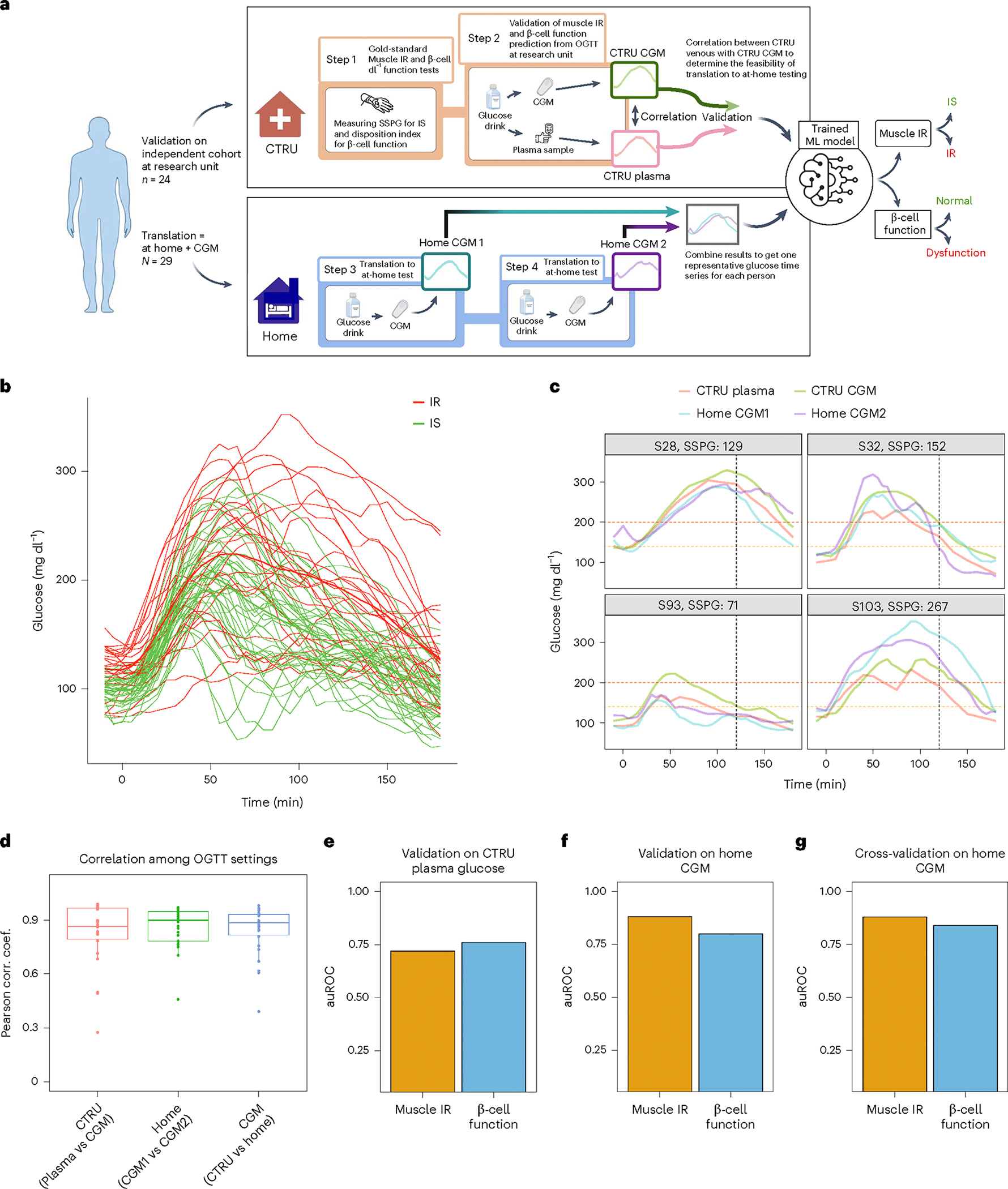
Validation on independent cohort and translation to at-home CGM testing. **a**, Study design of the validation cohort and at-home OGTT test via CGM to predict muscle IR and β-cell function. Participants underwent gold-standard testing at the research unit for insulin resistance (SSPG test) and B-cell function (16-point OGTT with C-peptide deconvolution adjusted for SSPG and expressed as DI) as described, as well as two OGTTs administered at home under standardized conditions during which glucose patterns were captured by a CGM within a single 10-day session (Dexcom G6 pro). **b**, CGM glucose curves between IR (red) and IS (green) during an OGTT, with the thicker line representing the mean curve. **c**, Examples of four participants, each with four glucose curves; two curves from OGTT at clinical setting via venous sampling and CGM, and two curves from at-home OGTT via CGM. Participants S28 and S93 are IS (SSPG < 120 mg dl^−1^), and participants S32 and S103 are IR (SSPG > 120 mg dl^−1^). The black vertical dashed line represents the 120-min timepoint when the glucose level was measured for clinical diagnosis of diabetes. A glucose value above 200 mg dl^−1^ (red dashed line) represents a diabetes status, glucose value below 140 mg dl^−1^ (orange dashed line) a normoglycaemic status, and glucose value between 140–200 mg dl^−1^ a prediabetes status. **d**, Correlations among glucose time series measured via different OGTT settings (clinical settings vs at-home, plasma vs interstitial via CGM, and reproducibility of 2 at-home OGTT via CGM). Each point represents the correlation between two glucose series of the same person. High positive correlations indicate that time-series patterns are preserved among test settings. The line inside each box represents the median Pearson correlation coefficient for each OGTT setting. The lower and upper edges of the box correspond to the 25th (Q1) and the 75th (Q3) percentiles of the data, respectively. **e**, Muscle IR and β-cell function prediction performance using plasma glucose series from the independent validation cohort during an OGTT at CTRU. **f**, Muscle IR and β-cell function prediction performance on CGM glucose series from the independent validation cohort during an at-home OGTT. **g**, Muscle IR and β-cell function prediction performance using cross-validation on CGM glucose series from the at-home cohort during an OGTT at home.

We next validated the prediction of muscle IR and β-cell function in a separate independent cohort of 24 individuals matched for age, sex, BMI and HbA1c (Fig. 5a, Table 1 and Methods). Frequently sampled OGTT as well as IST were conducted under standardized conditions in the CTRU as previously described to obtain gold-standard measures of muscle IR and β-cell function. The model focused on features extracted from glucose time series (OGTT_G_Features) as previously described and demonstrated in Fig. 3a. For each participant, muscle IR and β-cell function were predicted using the best-performing trained model from the initial cohort (Fig. 4 and Methods). On the independent validation cohort and using plasma glucose series obtained through OGTT at CTRU, muscle IR was predicted with an auROC of 0.72, and β-cell function with an auROC of 0.76 (Fig. 5e).

### At-home prediction of muscle insulin resistance and β-cell function through the use of continuous glucose monitoring

To demonstrate the practical value of performing this test at home as well as the reproducibility of our proposed framework, we sought to investigate the feasibility of utilizing at-home testing with CGM to predict muscle IR and β-cell function. Cohort characteristics of 29 individuals who were recruited from the main (*n* = 5) and validation (*n* = 24) cohorts are summarized in Table 1. Participants underwent gold-standard testing for insulin resistance (SSPG test) and B-cell function (16-point OGTT with C-peptide deconvolution adjusted for SSPG and expressed as DI) as described, as well as two OGTTs administered at home under standardized conditions during which glucose patterns were captured by a CGM within a single 10-day session (Dexcom G6 pro) (Fig. 5a and Supplementary Table 5).

Participants consumed a 75-g glucola (NERL Trutol, Thermo Fisher) in a 10-oz serving (identical to the method used in the CTRU OGTT) after an overnight fast of 10–12 h. They were instructed to follow dietary and physical activity guidelines to standardize the test to minimize environmental influences on glucose response (Methods). CGM curves for participants defined by SSPG as IR or IS are shown in Fig. 5b. Even with visual inspection of the curves, clear differences between IR and IS individuals are apparent: compared with IS, IR individuals had higher glucose peak (*P* < 0.05), a broader glucose curve that remained high, and thus higher AUC. This pattern is consistent with our previous findings (Fig. 3b) when plasma glucose levels were measured at various intervals in the CTRU.

**Extended Data Fig. 9 |:**
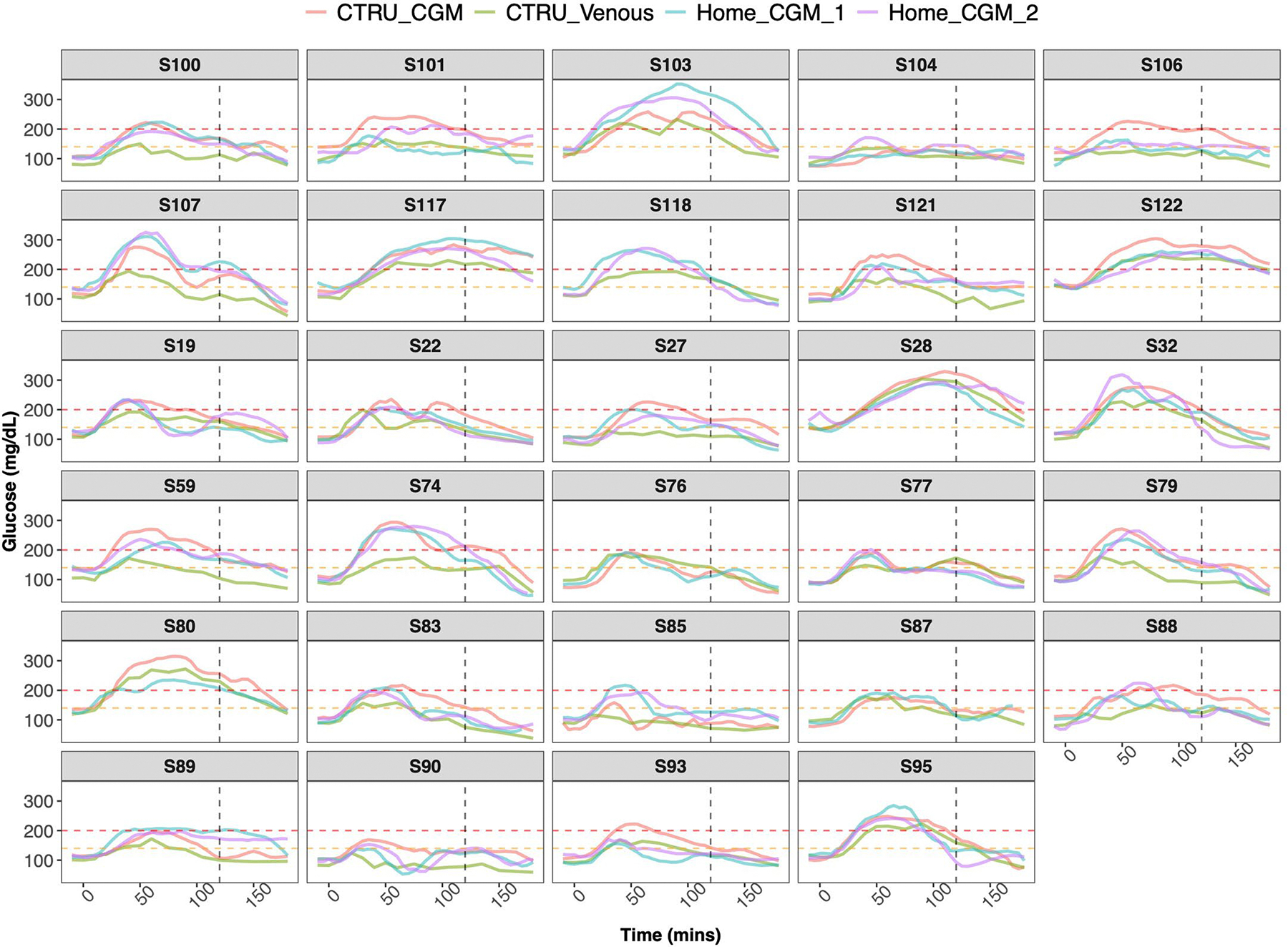
Glucose time-series from the validation and at-home CGM cohort. Each box represents one participant. Each participant has four glucose time-series; glucose time-series measured via venous blood sample during clinical OGTT (green), glucose time-series measured via CGM during the clinical OGTT (red), glucose time-series measured via CGM during 1st at-home testing trial (cyan), and glucose time-series measured via CGM during 2nd at-home testing trial (purple). Gold standard muscle insulin resistance is measured via SSPG, written on the header of each box.Time=0 on the x-axis represents the time when participants start drinking the glucose drink. The vertical black dashed line represents the time at 120 min when the glucose level is measured for clinical diagnosis of diabetes. A glucose value above 200 mg/dL (red dashed line) represents a diabetes status, a glucose value below 140 mg/dL (orange dashed line) represents a normoglycemic status, and glucose value between 140–200 mg/dL represents a prediabetes status.

We conducted a thorough investigation into the congruence of glucose time series acquired through different methods of OGTT, including (1) measurements of glucose levels from plasma samples collected after glucola consumption while simultaneously wearing a CGM and (2) in the home setting using CGM. Figure 5c displays a representative example of the glucose time series for four participants, each of whom had four glucose time series taken during different OGTT settings: two simultaneous series in the clinical research unit, one measured venously and the other via CGM, and two series acquired via two repeated at-home OGTT using CGM (see Extended Data Fig. 9 for all time series of all participants). Despite minor variations among the time series, the pattern of the glucose time series remained consistent across all settings. The concordance between different settings was quantified and depicted for each participant in Fig. 5d. In general, the Pearson correlation between the glucose time series obtained in the clinical setting through plasma and CGM measurements was 0.81 (*P* < 2.2 × 10^−16^); the correlation between the two at-home OGTT using CGM was 0.86 (*P* < 2.2 × 10^−16^); and the correlation between the clinical-setting time series measured venously and the mean of the two at-home OGTT time series was 0.80 (*P* < 2.2 × 10^−16^). These high correlations indicate that CGM measurements performed at home are similar to those obtained in the clinical research unit and have high concordance with each other when performed under standardized conditions to minimize environmental perturbations.

Features from the glucose time series curves were extracted as previously described in Fig. 3a. The classification of muscle IR (auROC = 0.88) and β-cell function (auROC = 0.80) based on the mean of two home CGM curves performed even better than the classification based on plasma curves in the independent test set (Fig. 5f). This improved performance is probably due to the efficient information extraction from both the feature engineering and the continuous measurements of CGM, which contains much more information than those from the venous sampling in which measurements are less frequent. A cross-validation based on CGM data alone showed similar performance for muscle IR (auROC = 0.88), whereas the prediction of β-cell function was further improved (auROC = 0.84) (Fig. 5g).

## Discussion

T2D and its precursor, prediabetes, are currently defined on the basis of glycaemic level rather than underlying pathophysiology. However, the mechanistic basis is complex and several distinct pathophysiological processes contribute. Here we demonstrate that individuals with glucose ranging from normoglycaemic to prediabetes exhibit clear heterogeneity in four distinct physiologic processes that contribute to disordered glucose metabolism, including muscle IR, β-cell dysfunction, impaired incretin effect and hepatic IR. We clearly showed that these phenotypes do not necessarily correlate with traditional glucose measures such as HbA1c and thus cannot be estimated in this way. Using gold-standard physiologic tests and expressing values for each individual as the relative deviance from the cohort mean, we expressed the degree to which each process was abnormal. The majority of individuals exhibited a single dominant metabolic subphenotype or two co-dominant phenotypes. These partitioned generally into IR dominant subphenotypes (muscle and liver IR), comprising 34% of the cohort, or insulin secretion subphenotypes (B-cell and incretin deficient), comprising 40% of the cohort. Muscle and hepatic IR were highly intercorrelated and frequently exhibited co-dominance. This suggests that from a clinical classification perspective, muscle and hepatic IR might be combined into one group classified as ‘insulin resistant’, and therapies known to improve insulin resistance (such as dietary or pharmacologic weight loss (including GLP-1 or dual GLP-1/GIP agonists), physical activity, metformin, thiazolidinediones) could be used as initial treatment. β-cell and incretin deficiency phenotypes, together accounting for the majority of patients, might be amenable to similar treatment strategies that target insulin secretion since this is the common denominator in the pathophysiology for both of these subphenotypes. These include agents that augment or replace insulin secretion or incretin action (for example, sulfonylureas, glinides, DPP4 inhibitors, GLP-1 agonists or GLP-1-GIP dual agonists, or insulin). Only 9% of participants could not be classified as having a dominant or co-dominant metabolic subphenotype, thus indicating that use of the glucose ‘shape of the curve’ method to identify underlying physiology could inform targeted approaches for diabetes prevention or treatment.

Historically, subclassification of T2D into more-specific subtypes has been limited by the lack of feasible scalable standardized diagnostic tests. The requirement for laborious and invasive research tests to characterize underlying metabolism precludes their use outside of a research setting, and surrogates such as HOMA-IR only explain 40% of insulin resistance^41^ and are limited by lack of standardization of insulin concentrations across laboratories. Recent advances in understanding the underlying biology of glycaemic disorders and the development of pharmacologic therapies targeting multiple distinct pathways to hyperglycaemia render a physiologically based subclassification of prediabetes and T2D more important than ever. In fact, the scientific discovery regarding the ‘incretin’ effect has led to the development of an entire new class of effective pharmacotherapies for T2D. Other classes of glucose-lowering therapeutics, as well as dietary weight loss and exercise target insulin resistance, β-cell function, hepatic glucose production and urinary glucose reabsorption. The majority of these medications and lifestyle interventions have been shown not only to reduce glucose in T2D, but also to significantly reduce the risk of progression to T2D in individuals with prediabetes^14–16,18,50^. Thus, if pathophysiology could be identified in each individual, targeted therapy and a precision-medicine approach would be possible. Given that 95% of the 13 M individuals with diabetes are grouped into one category of T2D, and the availability of an increasing array of therapeutic options, it is timely to consider subclassifying T2D. However, previous attempts to do so based on simple laboratory tests or clinical/demographic markers that ‘cluster’ together do not lend themselves to a true precision approach to treatment, which is based on targeting underlying physiology. Thus, the shape of the glucose curve using longitudinal measures and a machine-learning approach represents successful subclassification according to underlying physiology and warrants testing in trials that evaluate the relative efficacy of targeted treatment. Our study starts with physiological subphenotypes and then seeks a biomarker (shape of the glucose curve) that could be used in the clinic and at home.

A prerequisite for physiologic subclassification is a practical, accurate, accessible and standardized test. Gold-standard tests for insulin resistance, insulin secretion and incretin effect entail the use of intravenous infusions in the research unit and are laborious, invasive and expensive, hence are neither scalable nor practical. Thus, we seek to validate a more practical measure such as the shape of the glucose curve, particularly obtained by CGM, that could be used in the clinical setting. The OGTT test is relatively more feasible and has been widely studied with regard to its potential not only to identify diabetes on the basis of a 2-h glucose, but also to predict the risk of developing T2D, CVD and total mortality^30,31^. Furthermore, glucose obtained at 2 to 5 timepoints during OGTT has been correlated in multiple studies with underlying physiology^30,32–37^. Simple measures such as 1-h glucose peak, time to peak, nadir below baseline, or relative height of 2 vs 1 h or baseline glucose were noted to correlate with insulin resistance and β-cell function^32–34^. A number of publications showed that a mono versus biphasic curve shape was associated with insulin resistance, β-cell dysfunction and risk for diabetes^35–37^. Due to the simplicity of these analyses and the risk of missing the glucose peak with limited sampling and the high false-positive rate given that the majority of normoglycaemic individuals have a monophasic curve, more sophisticated modelling approaches have been employed^31,37,51^.

Functional principal component analysis (PCA) in pregnant women was able to differentially predict development of gestational diabetes^51^, and latent class trajectory analysis^37^ identified four curve types which differed according to degree of insulin resistance and β-cell function, and predicted diabetes, cardiovascular events and total mortality^31^. Results of the current study extend those of the previous studies by applying a machine-learning framework to analyse the dynamic glucose time series response to an OGTT with 16 timepoints, with demonstrated ability to identify insulin resistance, β-cell defect and incretin defect better than currently available tests including surrogate biomarkers, clinical-demographic profile and polygenic risk score. This machine-learning framework did not predict hepatic IR any better than HOMA-IR. However, given the high correlation between muscle and hepatic IR, we propose that it may be prudent to consider these two IR subphenotypes as a single IR phenotype. Despite the increasing potential of sophisticated approaches to leverage the OGTT to predict underlying metabolic phenotype, the OGTT test still poses a patient burden, limiting its widespread clinical use.

In the current study, we aimed to evaluate whether a glucose curve generated by CGM would yield a similar ability to identify underlying metabolic physiology. Such test can be performed conveniently at home, is facile and inexpensive. Our results show that when applied to a CGM-generated glucose curve following standardized OGTT, the same machine-learning algorithm can accurately identify insulin resistance. The best performance was obtained by using the average of two home OGTTs performed in a single week under standardized conditions. Indeed, the average of two home CGM curves after OGTT performed better than plasma glucose values obtained following OGTT in the CTRU, presumably due to the high measurement frequency. Glucose curve features from at-home OGTT via CGM were extracted similarly to those from the frequently sampled OGTT in CTRU (Fig. 3a). We have tested the percent in range and percent out of range as possible features^52–55^; however, these did not increase model performance. This is mainly because we used them during only a 180-min session and not the whole 10-day session. Also, percent in range and percent out of range are good for people with diabetes who usually have elevated glucose (hyperglycaemia), or who are taking insulin and exhibit hypoglycaemia. Our cohort is mainly normoglycaemic and people with prediabetes. One major concern about the OGTT is the previously noted intra-individual variability^56^ of glucose at a given timepoint. This is in part likely but not completely due to environmental stressors (for example, eating a late dinner or snack, eating out, alcohol, late exercise or excessive duration of fasting, morning stress or coffee, change of location or time, or rapidity of consumption of OGTT). We controlled for these with detailed patient instructions, and intra-patient variability was lowest for the home OGTT where the coefficient of variation was 11% for CGM across all timepoints. This compares favourably to the published 16.7% for 2-h plasma glucose during OGTT^56^. The high reproducibility of home CGM may also be due to similar conditions that can be maintained as well as the fact that we evaluated variability for the shape of the curve rather than a single timepoint, the latter of which may be more subject to variability, as we noted that some curves were of similar shape but shifted up, down, right or left compared with the other curve obtained from the same participant. If this method moves to the clinic for widespread use, it will be important to minimize environmental stressors that lead to variability in glycaemic responses to the OGTT, which can be accomplished with simple instructions for use.

Limitations of the current study include the relatively homogeneous demographics, largely comprising middle-aged Caucasians. Our BMI and glycaemic ranges were sufficiently broad. Further studies to examine differences in predictive potential based on race, ethnicity and sex, as well as age and broader BMI subgroups would be informative. The current results do not extend to T2D beyond very mild cases with HbA1c of 6.5% or below (diet-controlled or new-onset mild diabetes). Our two patients with diabetes were not previously diagnosed and had HbA1c of 6.5% and normal fasting glucose, hence are not reflective of more advanced diabetes. Additional studies will be required to determine whether this method can be applied to individuals with T2D with more advanced hyperglycaemia. At present, it seems the greatest utility of this method will be to diagnose the underlying physiologic subphenotype in individuals at risk for diabetes. Also of note, we used a 3-h OGTT and the results may be different when compared with a 2-h OGTT. Second, while the reproducibility of the OGTT is historically imperfect, we showed high correlation between the two CGM curves generated during home OGTT, with an acceptable coefficient of variation (11%), probably due to our efforts to ensure standardization of environmental conditions. It will be important to have two of these done under standardized conditions and if one is vastly different from the other, a third should be done and the outlier excluded due to a presumed environmental confounder. The optimal number of required at-home tests can be determined in future studies. In addition, the hepatic IR index used in this study is based on a formula that was validated against a gold-standard test rather than being a gold-standard test itself^47^. Thus, it is less precise than our other metabolic measures, which are highly precise. This probably explains the suboptimal performance in predicting hepatic IR from glucose time series, compared with HOMA-IR or the Matsuda index.

In summary, we demonstrate that the metabolic physiology underlying glycaemic disorders varies widely between individuals and that dominant or co-dominant metabolic subphenotypes exist, which has implications for precision approaches to treat/prevent T2D given the existence of interventions that target the separate physiologic components. We further show that modelling a glucose time-series response to oral glucose challenge can identify these metabolic subphenotypes. The features of the glucose time series can identify muscle IR, β-cell deficiency and incretin deficiency with superior predictive power compared with standard clinical and laboratory measures, accepted plasma surrogate markers and polygenic risk score. We further show that prediction is even stronger using the average of two CGM glucose curves generated during home OGTT administered under controlled conditions to minimize environmental confounding. We thus suggest that identification of distinct metabolic subphenotypes using features of the glucose time series during OGTT, including home OGTT with CGM, may enhance early identification of at-risk individuals who can then undergo targeted pharmacologic and lifestyle modification to prevent T2D. These results demonstrate how new technologies can unleash the potential of classic tests for metabolic health. If the patient burden posed by multiple blood draws over a 3-h window is removed, the increased practicality and feasibility of OGTT at home may yield a metabolic test suitable for widespread use to identify metabolic phenotypes for risk stratification and targeted treatment. Thus, the current results provide support for the use of CGM to define metabolic subphenotypes on the basis of the shape of the glucose curve during OGTT, and warrant trials to evaluate the relative efficacy of targeted treatment based on identified subphenotypes.

## Methods

This study is part of the Precision Diets for Diabetes Prevention clinical trial (NCT03919877).

### Study participants

The study protocol and clinical investigation were approved by the Stanford University School of Medicine Human Research Protection Office (Institutional Review Board #43883). Participants provided written informed consent. Participants were recruited from the San Francisco Bay Area via locally placed advertisements online, in local newspapers and during faculty lectures to the community. Participants were screened with a medical history and physical examination in the Stanford University Clinical and Translational Research Unit (CTRU), where vital signs, fasting blood plasma glucose and baseline haematocrit, alanine aminotransferase (ALT) and creatinine were obtained. Eligibility criteria included age of 30–70 years, BMI of 23–40 kg m^−2^, absence of major organ disease, absence of type 2 diabetes defined by personal history or fasting glucose ≥126 mg dl^−1^, uncontrolled hypertension, malignancy, chronic inflammatory conditions, use of any medications known to alter blood glucose or insulin sensitivity, haematocrit <30, creatinine above the normal range and ALT >2-fold above the upper limit of the normal range.

#### Initial cohort.

A total of 36 participants enrolled and 32 completed all metabolic tests and were included in the final analyses as the main study cohort (also called training cohort) (Table 1 and Supplementary Table 1).

#### Validation cohort.

An independent cohort of 24 individuals completed OGTT and IST in the CTRU, as well as measurement of HbA1c and lipid panel.

#### At-home CGM cohort.

The validation cohort plus 5 of the initial cohort (*n* = 29) was used for CGM analysis both in the CTRU (they were measured simultaneously during the OGTT test with plasma sampling and a blinded Dexcom G6 CGM), and two at-home glucose-tolerance tests on separate mornings under standardized conditions (see below).

### Quantitative metabolic physiological tests

All tests were performed in the Stanford CTRU after a 10-h overnight fast.

#### (1) Frequently sampled OGTT.

Participants were instructed to avoid celebratory or restrictive eating and unusual physical activity for 3 days before performing the test, fast 10–12 h before the test, have no strenuous exercise after 17:00 the evening before the test and no smoking or using a nicotine patch the morning of the test. Dynamic plasma glucose profiles were obtained after administration of a 75 g oral glucose load over 5 min (Brand Trutol, Thermo Fisher, 75 g in a 10-oz serving). Plasma samples were drawn from an antecubital intravenous catheter drawn at 16 timepoints (−10, 0, 10, 15, 20, 30, 40, 50, 60, 75, 90, 105, 120, 135, 150 and 180 min). Glucose level was measured at the 16 timepoints using the oximetric method; insulin and C-peptide using the Millipore radioimmunoassay assay, both at 7 timepoints (0, 15, 30, 60, 90, 120 and 180 min); GLP-1 using the Millipore ELISA EZGLP1T-36K kit, GIP using the Millipore ELISA EZHGIP-54K kit, and glucagon using the Mercodia ELISA 10–1271-01 kit, all at 4 timepoints (0, 30, 60 and 120 min). Insulin, C-peptide, GLP-1, GIP and glucagon were measured at the Core Lab for Clinical Studies, Washington University School of Medicine in St Louis. From the baseline sample (at 0 min), HbA1c, fasting plasma glucose, insulin, triglyceride and HDL cholesterol levels were determined.

#### (2) At-home OGTT with CGM.

Participants wearing CGM were instructed to do two at-home OGTTs that were standardized to minimize environmental influences that might lead to variability in results. The two at-home OGTTs were performed within a single CGM sensor session (10 days).

After an overnight fast, a 75-g glucola drink (NERL Trutol, Thermo Fisher) was consumed in 10 oz over 5 min and for 3 h, no oral intake other than water was allowed, and the patients remained seated, identical to the OGTT procedure performed in the CTRU. Participants were required to do this test during a typical week that was devoid of celebratory or restrictive eating (for example, wedding or religious holiday), with usual activity patterns (no unusual activity/training outside their typical patterns); to refrain from eating out, performing strenuous exercise after 17:00 the night before the test, eating or consuming anything other than water after 22:00 on the evening before and morning before the test; and to avoid smoking or using a nicotine patch the morning of the test.

#### (3) Isoglycaemic intravenous glucose infusion.

IIGI, as previously described^46^, was performed to quantify the incretin effect by duplicating the plasma glucose profile during the corresponding OGTT. During the IIGI test, an intravenous catheter was placed in the antecubital vein for administration of a continuous dextrose infusion at a rate needed to obtain the desired glucose, which for each timepoint was the glucose level obtained at the same timepoint during the OGTT. Blood sampling from a second intravenous catheter included the same timepoints and assays as described above for the OGTT.

#### (4) Insulin suppression test.

Insulin-mediated glucose disposal was quantified by the modified insulin suppression test as originally described and validated^38–40^. Briefly, participants were infused for 240 min with octreotide (0.27 μg m^−2^ min^−1^) (to suppress endogenous insulin secretion), insulin (32 mU m^−2^ min^−1^) and glucose (267 mg m^2^ min^−1^). Blood was drawn at 30-min intervals for monitoring and at 10-min intervals from 210 to 240 min of the infusion to measure plasma glucose and insulin concentrations, and the mean of these four glucose values (210, 220, 230, 240 min) was used as the steady-state plasma insulin (SSPI) and glucose (SSPG) concentrations for each individual. As SSPI concentrations were similar in all participants during these tests, the SSPG concentration provided a direct measure of the ability of insulin to mediate disposal of an infused glucose load; the higher the SSPG concentration, the more insulin resistant the individual.

#### (5) Measurement of plasma glucose.

Blood was drawn from an intravenous catheter placed in the antecubital fossa which was warmed with a heating pad to obtain arterialized venous blood. The blood sample was run to the CTRU lab, spun for 2 min and plasma was analysed on the glucose analyzer (YSI 2500) calibrated to the clinical laboratory glucose values at our institution. This method allowed for immediate bedside glucose results so that for the IIGI (above), the glucose infusion rate could be altered every 5 min as needed to mimic the glucose curve generated by the OGTT.

### Metabolic calculations

#### (1) Muscle insulin resistance (SSPG).

While SSPG as described above reflects whole-body insulin-mediated glucose disposal, 85% of this occurs in muscle^7^ and thus, to distinguish this measure from hepatic insulin resistance, the term ‘muscle’ insulin resistance is used in this study. Furthermore, a cut-off of <120 mg dl^−1^ is generally indicative of insulin sensitivity. For the purpose of this paper, we defined non-insulin sensitivity of individuals (those with SSPG ≥ 120 mg dl^−1^) as insulin resistance (IR). This includes individuals with moderate and severe IR, all of whom have been shown to respond to treatments known to improve insulin sensitivity (both lifestyle and medications).

#### (2) β-cell function (DI).

To quantify the status of β-cell function, we first calculated insulin secretion rate using the C-peptide deconvolution method, using C-peptide concentrations measured during OGTT tests at 0, 15 and 30 min. The Insulin SECretion (ISEC) software^42^ was used to calculate prehepatic insulin secretion from plasma C-peptide measurements with adjustment for age, sex and BMI^43^. The ISEC software can be obtained from the author Roman Hovorka of the Metabolic Modelling Group, Centre for Measurement and Information in Medicine, Department of System Science, City University, London, United Kingdom. We required an error coefficient of variation of 5% and 15-min intervals. As the programme uses a population model of C-peptide kinetics, we classified individuals as diabetic (‘niddm’) if they had either fasting plasma glucose >126 mg dl^−1^, 2-h glucose during OGTT >200, or HbA1c >6.5; or as ‘obese’ if they were non-diabetic with BMI > 30. The remaining individuals were classified as ‘normal’. Because insulin secretion is best interpreted relative to insulin resistance (insulin-resistant individuals need to secrete more insulin to maintain normoglycaemia), we then calculated DI^44,45^, as previously described, which adjusts the insulin secretion rate for degree of insulin resistance to generate an overall measure of B-cell function. DI was calculated as the area under the insulin secretion rate, divided by the SSPG (equation 1). DI thus describes β-cell function relative to insulin resistance.


(1) $$DI=\left(\frac{\int_{t=0}^{t=30}Insulin\;\text{Secretion}\;{\text{Rate}}_{t}^{OGTT}dt}{\text{SSPG}}\right)$$

#### (3) Incretin effect (IE%).

We quantified the incretin effect as previously described^46^ using equation (2). IE was calculated on the basis of the difference in the C-peptide concentrations during a frequently sampled OGTT and IIGI (0–180 min) that by design induce nearly identical plasma glucose concentrations. The difference in C-peptide in response to oral versus intravenous glucose loading reflects the contribution of incretin hormones from the gut that stimulate pancreatic β-cell insulin secretion. A higher incretin effect may indicate either increased secretion of incretin hormones (for example, GLP-1 and GIP) and/or β-cell responsiveness to these hormones. Lower incretin effect may be due to deficiency in incretin hormone secretion and/or relative inability to stimulate insulin secretion from the β cell.


(2) $$IE\%=\left(\frac{\int_{t=0}^{t=180}{\text{cpeptide}}_{t}^{OGTT}dt-\int_{t=0}^{t=180}{\text{cpeptide}}_{t}^{IIGI}dt}{\int_{t=0}^{t=180}{\text{cpeptide}}_{t}^{OGTT}dt}\right)\times 100$$

#### (4) Hepatic IR index.

Liver insulin resistance was inferred using a surrogate index as shown in equation (3) (ref. 47), where insulin is measured in (microIU ml^−1^), plasma HDL cholesterol is measured in (mg dl^−1^) and BMI is measured in kg m^−2^. In adults, body fat % (BF%) can be estimated using the Deurenberg Index as shown in equation (4)^57^, where BMI is measured in kg m^−2^, age in years, and sex = 1 for male and sex = 0 for female.


(3) $$\text{Hepatic}\;\text{IR}=\;-0.091+0.4\times log\left(\int_{t=0}^{t=180}{insulin}_{t}^{OGTT}dt\right)\;\;+0.346\times log(BF\%)-0.408\times log(HDL)+0.435\times log(BMI)$$

(4) BF%=-5.4+1.2×BMI+0.23×Age-10.8×Sex

### Quantifying the relative deviance for each physiologic measure for metabolic subphenotype assignment

To classify each participant to their most ‘abnormal’ physiologic process, we calculated a standardized deviance score (that is, a *Z*-score) for each metabolic measure for each participant (equation 5), where ${msp}_{j}^{i}$ is the *i*th metabolic measure of participant j and $mean\left({msp}^{i}\right)$ is the average of *i*th metabolic measure among the cohort’s participants and $sd\left({msp}^{i}\right)$ is the standard deviation. High positive deviance indicates a more abnormal score and stronger phenotype for that measure in a given individual; high negative deviance indicates a healthier metabolic measure, and thus relative absence of the unhealthy phenotype as compared with the average population. DI and IE% were negated before applying equation (5), since higher DI and IE% indicate healthier β-cell function and incretin function, respectively. As shown in the heat map in Fig. 2, it is possible to see in a given individual the relative deviance for each metabolic measure: some individuals had a single dominant (most deviant) measure, whereas others had two or more that were equally dominant.

(5) $$deviance({msp}_{j}^{i})=\frac{{msp}_{j}^{i}-mean({msp}^{i})}{sd({msp}^{i})}$$

where i∈{MuscleIR,BetaCellDysfunction,IncretinDysfunction,HepaticIR}.

For each participant, the dominant metabolic subphenotype was identified on the basis of the one exhibiting the highest deviance score. When the difference in deviance scores between the two highest metabolic subphenotypes was less than 0.5, the participant was classified as having co-dominant subphenotypes. If more than one subphenotype exhibited deviance scores within 0.5 of the highest score, the participant was classified as having neither dominant nor co-dominant subphenotypes. This method is not intended for the clinical diagnosis of metabolic abnormalities. Instead, it focuses on identifying individuals with deviations in metabolic profiles relative to a reference cohort. Further validation in diverse populations is required to assess its generalizability.

### Genotyping and T2D polygenic risk score calculation

DNA was extracted from whole-blood peripheral blood mononuclear cells (PBMC). The concentration of the extracted DNA was measured using Qubit, normalized to 50 ng μl^−1^, and 10 μl was used for each sample for genotyping. All samples were genotyped using the Illumina Infinium Omni2.5Exome array with ~2.6 million single nucleotide variants (SNV) sites measured. Genotyping data underwent standard quality control using PLINK^58^, including filtering of SNVs with excess missing genotypes (>2%), filtering of SNVs out of Hardy–Weinberg equilibrium (*P* < 1 × 10^−6^) and removal of samples with either excess missing genotypes (>3%) or discordant genetic vs recorded sex. Coordinates were aligned to the positive strand on genome build GRCh38 and the data were imputed against the TOPMed R2 reference panel^59–61^. After quality control and imputation, 11.8 million high-quality SNVs (*R*^2^ > 0.3) were obtained. T2D polygenic risk score (PRS_T2D_) was generated as an additive model using 338 index variants and corresponding weights from the DIAMANTE multi-ancestry meta-analysis study^49^. Of the 338 variants, 16 were not available, for which mean population scores were added using the TOPMed Bravo platform reference frequencies (2 × weight × population allele frequency)^61^.

### Machine-learning framework to identity metabolic subphenotypes from OGTT glucose time series

#### Preprocessing.

Glucose time series were preprocessed by imputing missing data using linear interpolation.

#### Features extraction.

We extracted features for the machine-learning framework using two feature extraction approaches (1) OGTT glucose time-series features (OGTT_G_Features) and (2) reduced representation of the OGTT glucose time series (OGTT_G_ReducedRep). In the first approach (OGTT_G_Features), we extracted 14 features from the imputed OGTT glucose time series, such as glucose level at time 0 (G_0_), 60 (G_60_), 120 (G_120_), 180 (G_180_), peak glucose level (G_Peak), length of the glucose time-series curve over the frequently sampled OGTT time interval (CurveSize), AUC, pAUC, negative (n)AUC, iAUC, coefficient of variation (CV), time from baseline to peak value (T_baseline2peak), slope between baseline to the peak glucose level (S_baseline2peak), and slope between glucose values at the peak and at the end (at *t* = 180 min) (S_peak2end). These 14 features demonstrated statistical significance in their Pearson correlation coefficients with one or more metabolic subphenotype indicators (SSPG, DI, IE, hepatic IR index). The only exception was ‘S_baseline2peak’, which exhibited a near-significant correlation with β-cell function (*P* = 0.06). In the second approach (OGTT_G_ReducedRep), we first *Z*-normalized the imputed OGTT glucose time series to extract features related to curve patterns rather than the amplitude, then we smoothed the *Z*-normalized glucose time series using a cubic smoothing spline with a smoothing parameter of 0.35. Then, the data were split into a training and testing set. For the testing set, we extracted the reduced representation of the OGTT glucose time series using PCA and the top two principal components were extracted to be used in training machine-learning classifiers (OGTT_G_ReducedRep). For each new glucose series in the test set (X), the series was centred first using the mean vector of the training data ($X_{\text{centred}}$). The dimension of the mean vector is 1 × 16, since we had 16 glucose timepoints per series. Afterwards, the centred series was multiplied by the loading matrix (W) of the top two principal components extracted from the training set (equation 6). The dimension of W is 16 × 2. $X_{\text{reduced}}$ is the projected test series on the reduced representation space of the training set.


(6) $$X_{\text{reduced}}=X_{\text{centred}}\times W$$

#### Training machine-learning models.

We next developed a machine-learning approach, where the two extracted sets of glucose time-series features (OGTT_G_Features and OGTT_G_ReducedRep) were tested independently. For each feature extraction approach, the dataset was split into a training set and a test set. Data were stratified on the basis of metabolic subphenotype class to ensure equal distribution of each class between training and test set. Participants from the initial venously sampled OGTT collection at CTRU without CGM data were assigned to the training set. No participants or samples existed in both training or test sets. Cross-validation was implemented on the training set.

For each metabolic subphenotype, we optimized the hyperparameters of 4 different classifiers: support vector machine with a radial basis function kernel (SVM-RBC), support vector machine with a linear kernel (SVM-linear), logistic regression with L1 regularization (LR-L1) and random forest (RF). We used the sklearn Python package to train, tune and test machine-learning models. LR-L1 was trained using a SAGA solver and run for a maximum of 10,000 iterations to converge, with a tunable regularization strength hyperparameters C. SVM-RBF and SVM-linear were trained using the sklearn.svm.SVC method, with kernel ‘rbf’ and ‘linear’, respectively. The regularization parameter ‘C’ is the tunable hyperparameter of both SVM models (linear and RBF). The tunable hyperparameters for random forest is the number of estimators. We had a grid of tunable hyperparameters (5 values) and searched the best one on the basis of cross-validation. All models were controlled by a preset random seed. The total number of models was 20 (4 basic model architectures and 5 hyperparameter values for each model).

Throughout the manuscript, all machine-learning experiments were conducted using 5-fold cross-validation and iterated 100 times. This entailed dividing the training set of plasma glucose from OGTT at CTRU (32 participants) into 5 groups, where 80% was used for model training and 20% was used for validation during each fold. In each fold, we calculated the auROC together with other metrics. To ensure a more rigorous evaluation and stable model, we repeated the above cross-validation 100 times, where we shuffled the data before dividing the data into folds. This ensures that the performance of the framework is generalizable to any division of the data. Different hyperparameters were tested for each model in cross-validation and the order was shuffled after each iteration. The final model with the selected hyperparameter value was chosen on the basis of best auROC in cross-validation. The process of hyperparameter tuning and model selection was merged in the same loop because of the limited training sample size. However, the shuffle process ensured an accurate estimation of performance. The model was then tested on the independent test set on both data (plasma glucose from OGTT at CTRU and CGM data), similar to the training set. The class imbalance problem was handled via stratified sampling to ensure that training and validation contained the same proportion of class samples. Importantly, in the second approach of feature extraction (OGTT_G_ReducedRep), reduced representation was obtained from the training set, not the whole dataset, and used to train each classifier. The test set was then projected onto the reduced representation space obtained from the trained dataset, and their representations were used to predict metabolic subphenotypes. Obtaining reduced representation space from the training dataset instead of the whole dataset was crucial for the robustness and generalizability of the model by preventing data leakage between the training dataset and the test dataset.

We then conducted a comprehensive experiment to predict metabolic subphenotypes using the two sets of extracted features as previously discussed (OGTT_G_Features and OGTT_G_ReducedRep). For comparison, we evaluated the performance of these two feature sets to surrogate markers for metabolic phenotypes that are in current use, including (1) demographics (age, sex, ethnicity, BMI, family history of T2D), (2) T2D polygenic risk scores, (3) incretins (total GIP and GLP-1 concentration at hour 2 of OGTT_2h), (4) laboratory (HbA1c and FPG), (5) HOMA-B^62^, (6) HOMA-IR^62^ and (7) Matsuda Index^63^. Demographics was evaluated alone and in all other models including our OGTT_G_Features and OGTT_G_ReducedRep models.

The performance of each model on each feature set and on each metabolic subphenotype was evaluated using auROC. We also calculated sensitivity, specificity, F1, precision and accuracy. Metrics were aggregated and summarized.

#### Model validation on an independent cohort of OGTT at the research unit.

The independent validation cohort was first preprocessed, then used to extract the two sets of glucose time series features similarly to the initial cohort. Then, trained models from the initial cohort were used to predict muscle IR.

### Statistical analyses

Statistical significance was performed using Wilcoxon rank-sum test with Bonferroni correction when multiple testing was required, such as the case with evaluating the metabolic subphenotype predictions using different feature sets. All *P* values were Bonferroni corrected.

### Visualization

We used the ggplot2 (v.3.3.2) and pheatmap (v.1.0.12) R packages to plot most of the figures. Figures 1a and 5a were created using the Figma design tool (https://www.figma.com/). Figure 3a was created using the draw.io platform (https://app.diagrams.net).

### Reporting summary

Further information on research design is available in the Nature Portfolio Reporting Summary linked to this article.

## Extended Data

## Supplementary Material

Supp8

Supp7

Supp6

Supp4

Supp5

Supp3

Supp2

Supp1

Reporting Summary

## Figures and Tables

**Table 1 |:** Baseline characteristics of the study cohorts

Participant characteristics	Initial cohort (*N* = 32)	Validation cohort (*N* = 24)	At-home CGM cohort (*N* = 29)
Age, years	57.2 ± 8.5	53.2 ± 10.4	54.9 ± 10.4
Sex, M/F	13/19	11/13	14/15
BMI, kgm^−2^	26.5 ± 4.2	25.9 ± 5.1	25.6 ± 4.8
Ethnicity/race, Caucasian/Asian/Hispanic/African American	23/8/1/0	17/7/0/0	20/8/1/0
Systolic blood pressure, mmHg	113 ± 11.4	121.5 ± 12.7	121.5 ± 13.8
Diastolic blood pressure, mmHg	70 ± 8.5	73.3 ± 9.6	73.3 ± 10.1
HbA1c, %	5.6 ± 0.4	5.6 ± 0.5	5.6 ± 0.5
Glycaemic status (based on HbA1c), normoglycaemic/preDM/T2D	17/14/1	16/7/1	18/9/2
Fasting plasma glucose, mg dl^−1^	97.8 ± 12.4	97.2 ± 14.2	98.4 ± 14.4
2-h glucose during OGTT, mg dl^−1^	130.8 ± 34.7	136.9 ± 52.7	142.1 ± 57.1
Total cholesterol, mg dl^−1^	194.8 ± 36.9	177.8 ± 40.8	176.1 ± 38.3
Triglyceride, mg dl^−1^	90.1 ± 41.9	92.4 ± 52.5	87.8 ± 50.1
HDL, mg dl^−1^	66.3 ± 22.7	58.0 ± 22.3	59.1 ± 21.9
LDL, mg dl^−1^	110.5 ± 29.6	102.7 ± 32.7	100.6 ± 30.2
Fasting insulin, microIU ml^−1^	9.9 ± 7.1	11.5 ± 9.9	10.8 ± 9.2
hs-CRP, mg l^−1^	1.3 ± 1.2	1.95 ± 1.9	1.7 ± 1.8
ALT, Ul^−1^	28.4 ± 11.8	27.8 ± 16.1	26.8 ± 15.2

Continuous variables are reported as average ± s.d. and categorical variables as frequency. LDL, low-density lipoprotein; hs-CRP, high-sensitivity C-reactive protein; ALT, alanine aminotransferase.

**Table 2 |:** Performance metrics of the best models for each metabolic subphenotype for each feature set

Prediction task	Feature set	auROC	Sensitivity	Specificity	F1	Precision	Accuracy	Classifier
Muscle IR	Demographics	0.37	0.55	0.56	0.55	0.58	0.56	SVM-RBF
	Demographics + PRS	0.21	0.38	0.26	0.27	0.25	0.29	RF
	Demographics + Lab	0.69	0.67	0.65	0.66	0.68	0.66	LR-L1
	Demographics + Incretins	0.38	0.85	0.34	0.66	0.58	0.59	SVM-RBF
	Demographics + HOMA-B	0.44	0.98	0.02	0.40	0.50	0.53	LR-L1
	Demographics + HOMA-IR	0.77	0.74	0.71	0.73	0.76	0.72	LR-L1
	Demographics + Matsuda	0.83	0.85	0.42	0.71	0.66	0.65	SVM-linear
	Demographics + OGTT_G_Curve_Features	0.90	0.90	0.75	0.83	0.81	0.82	SVM-linear
	Demographics + OGTT_G_ReducedRep	0.95	0.81	0.91	0.85	0.93	0.87	LR-L1
3-ceLL function	Demographics	0.49	0.69	0.30	0.52	0.60	0.51	SVM-RBF
	Demographics + PRS	0.46	0.77	0.12	0.35	0.42	0.44	LR-L1
	Demographics + Lab	0.50	0.82	0.18	0.59	0.62	0.56	SVM-linear
	Demographics + Incretins	0.72	0.93	0.01	0.54	0.43	0.53	SVM-RBF
	Demographics + HOMA-B	0.48	0.98	0.01	0.52	0.22	0.58	SVM-linear
	Demographics + HOMA-IR	0.57	0.71	0.19	0.55	0.55	0.50	LR-L1
	Demographics + Matsuda	0.44	0.74	0.29	0.61	0.62	0.56	SVM-RBF
	Demographics + OGTT_G_Curve_Features	0.84	0.78	0.72	0.79	0.82	0.75	SVM-linear
	Demographics + OGTT_G_ReducedRep	0.89	0.82	0.73	0.82	0.85	0.78	SVM-linear
Incretin effect	Demographics	0.62	0.51	0.88	0.63	0.81	0.72	LR-L1
	Demographics + PRS	0.53	0.33	0.67	0.37	0.41	0.54	RF
	Demographics + Lab	0.80	0.64	0.78	0.66	0.74	0.71	RF
	Demographics + Incretins	0.68	0.60	0.64	0.54	0.56	0.62	RF
	Demographics + HOMA-B	0.68	0.62	0.61	0.55	0.57	0.61	RF
	Demographics + HOMA-IR	0.75	0.60	0.76	0.63	0.71	0.69	RF
	Demographics + Matsuda	0.48	0.72	0.56	0.63	0.56	0.62	SVM-linear
	Demographics + OGTT_G_Curve_Features	0.90	0.75	0.74	0.71	0.73	0.74	SVM-RBF
	Demographics + OGTT_G_ReducedRep	0.88	0.79	0.72	0.73	0.71	0.75	LR-L1
Hepatic IR	Demographics	0.63	0.71	0.47	0.60	0.57	0.58	LR-L1
	Demographics + PRS	0.45	0.34	0.61	0.33	0.36	0.51	SVM-RBF
	Demographics + Lab	0.78	0.90	0.45	0.74	0.66	0.67	SVM-RBF
	Demographics + Incretins	0.73	0.70	0.60	0.63	0.62	0.64	RF
	Demographics + HOMA-B	0.50	0.52	0.48	0.48	0.50	0.48	SVM-RBF
	Demographics + HOMA-IR	0.88	0.80	0.80	0.78	0.83	0.80	LR-L1
	Demographics + Matsuda	0.90	0.82	0.71	0.76	0.78	0.76	SVM-linear
	Demographics + OGTT_G_Curve_Features	0.84	0.78	0.73	0.76	0.76	0.75	RF
	Demographics + OGTT_G_ReducedRep	0.54	0.73	0.52	0.65	0.64	0.63	SVM-linear

## Data Availability

The de-identified glucose values from CGM and venous, along with other data types used in this study, can be downloaded from the study data repository at https://storage.googleapis.com/gbsc-gcp-project-ipop_public/metabolic_subphenotype_db/metabolic_subphenotypes_db.zip.
