# Supplementary material for: Prediction of metabolic subphenotypes of type 2 diabetes via continuous glucose monitoring and machine learning
Source: Nat Biomed Eng. 2024 Dec 23;9(8):1222–39. doi: 10.1038/s41551-024-01311-6 (PMC12183321; doi:10.1038/s41551-024-01311-6)
Supplement: Supplementary file 1 — Reporting Summary [file 41551_2024_1311_MOESM1_ESM.pdf]

nature portfolio

Corresponding author(s): Tracey McLaughlin and Michael Snyder

Last updated by author(s): Oct 21, 2024

Reporting Summary

Nature Portfolio wishes to improve the reproducibility of the work that we publish. This form provides structure for consistency and transparency in reporting. For further information on Nature Portfolio policies, see our Editorial Policies and the Editorial Policy Checklist.

Statistics

For all statistical analyses, confirm that the following items are present in the figure legend, table legend, main text, or Methods section.

n/a	Confirmed	
☐	☒	The exact sample size (*n*) for each experimental group/condition, given as a discrete number and unit of measurement
☐	☒	A statement on whether measurements were taken from distinct samples or whether the same sample was measured repeatedly
☐	☒	The statistical test(s) used AND whether they are one- or two-sided<br>*Only common tests should be described solely by name; describe more complex techniques in the Methods section.*
☐	☒	A description of all covariates tested
☐	☒	A description of any assumptions or corrections, such as tests of normality and adjustment for multiple comparisons
☐	☒	A full description of the statistical parameters including central tendency (e.g. means) or other basic estimates (e.g. regression coefficient) AND variation (e.g. standard deviation) or associated estimates of uncertainty (e.g. confidence intervals)
☐	☒	For null hypothesis testing, the test statistic (e.g. *F*, *t*, *r*) with confidence intervals, effect sizes, degrees of freedom and *P* value noted<br>*Give P values as exact values whenever suitable.*
☒	☐	For Bayesian analysis, information on the choice of priors and Markov chain Monte Carlo settings
☒	☐	For hierarchical and complex designs, identification of the appropriate level for tests and full reporting of outcomes
☐	☒	Estimates of effect sizes (e.g. Cohen's *d*, Pearson's *r*), indicating how they were calculated

Our web collection on statistics for biologists contains articles on many of the points above.

Software and code

Policy information about availability of computer code

Data collection	The Insulin SECretion (ISEC) software was used to calculate insulin-secretion rate from plasma C-peptide measurements (PMID: 8894385).
Data analysis	R v4.1.0 and Python v3.6 were used for the statistical analyses, for machine-learning modeling, and for visualization. The codes are available at https://github.com/aametwally/Metabolic_Subphenotype_Predictor

For manuscripts utilizing custom algorithms or software that are central to the research but not yet described in published literature, software must be made available to editors and reviewers. We strongly encourage code deposition in a community repository (e.g. GitHub). See the Nature Portfolio guidelines for submitting code & software for further information.

Data

Policy information about availability of data

All manuscripts must include a data availability statement. This statement should provide the following information, where applicable:

- Accession codes, unique identifiers, or web links for publicly available datasets
- A description of any restrictions on data availability
- For clinical datasets or third party data, please ensure that the statement adheres to our policy

The de-identified glucose values from CGM and venous, along with other data types used in this study can be downloaded from the study data repository at https://storage.googleapis.com/gbsc-gcp-project-ipop_public/NBME-22-1010C/NBME-22-1010C.zip.

Research involving human participants, their data, or biological material

Policy information about studies with human participants or human data. See also policy information about sex, gender (identity/presentation), and sexual orientation and race, ethnicity and racism.

Reporting on sex and gender	We collected sex information of all participants in the study. We did not collect gender information. The findings apply to male and female.
Reporting on race, ethnicity, or other socially relevant groupings	Participant race and ethnicity data were based on self-reported information. The participant-recruitment process was conducted exclusively in the San Francisco Bay Area. Owing to the demographic characteristics of this region, the resulting cohort predominantly consisted of individuals identifying as Caucasian and Asian. Recognizing the potential influence of ethnicity on the study outcomes, it was methodically incorporated as a variable in our baseline prediction models for metabolic sub-phenotypes. Notably, all participants included in this study held a minimum educational level of a college degree.
Population characteristics	Three cohorts were used in this study; (1) a main cohort for training and testing the model, (2) a validation cohort, (3) an at-home CGM cohort. The main cohort of 32 participants (13 males and 19 females; average age 57.2 years, average BMI 26.5 kg/m^2, average HbA1c 5.6%). An independent validation cohort of 24 participants (11 males and 13 females; average age 53.2 years, average BMI 25.9 kg/m^2, average HbA1c 5.6%). At-home CGM cohort of 29 participants (24 from the validation cohort and 5 from the initial cohort; 14 males and 15 females; average age 54.9 years, average BMI 25.6 kg/m^2, average HbA1c 5.6%).
Recruitment	Participants provided written informed consent. Participants were recruited from the San Francisco Bay Area via locally placed advertisements online, in local newspapers, and during faculty lectures to the community.
Ethics oversight	The study protocol and clinical investigation were approved by the Stanford University School of Medicine Human Research Protection Office (Institutional Review Board # 43883).

Note that full information on the approval of the study protocol must also be provided in the manuscript.

Field-specific reporting

Please select the one below that is the best fit for your research. If you are not sure, read the appropriate sections before making your selection.

☒ Life sciences ☐ Behavioural & social sciences ☐ Ecological, evolutionary & environmental sciences

For a reference copy of the document with all sections, see nature.com/documents/nr-reporting-summary-flat.pdf

Life sciences study design

All studies must disclose on these points even when the disclosure is negative.

Sample size	The exact contribution of each metabolic sub-phenotype to glucose dysregulation was unknown before the start of the study. On the basis of our previous experience, we estimated that 30 participants would be sufficient to uncover heterogeneity in metabolic sub-phenotypes and to use the data to train machine-learning models. We managed to recruit 32 participants for the initial cohort, and another 24 as an independent cohort. The third cohort was for at-home CGM, and we recruited 29 participants (24 from the validation cohort and 5 from the initial cohort).
Data exclusions	No data were excluded.
Replication	The training was performed in a 4-fold cross-validation fashion and repeated 100 times. Hence, 75% of the initial cohort data were used for training and 25% in testing. An independent cohort was recruited to validate the trained models at the research unit and at home using CGM.
Randomization	Randomization was not applicable to the study.
Blinding	CGM data were collected in blinded mode, meaning that the participants did not see the real-time glucose values.

Reporting for specific materials, systems and methods

We require information from authors about some types of materials, experimental systems and methods used in many studies. Here, indicate whether each material, system or method listed is relevant to your study. If you are not sure if a list item applies to your research, read the appropriate section before selecting a response.

Materials & experimental systems

n/a	Involved in the study	
☒	☐	Antibodies
☒	☐	Eukaryotic cell lines
☒	☐	Palaeontology and archaeology
☒	☐	Animals and other organisms
☐	☒	Clinical data
☒	☐	Dual use research of concern
☒	☐	Plants

Methods

n/a	Involved in the study	
☒	☐	ChIP-seq
☒	☐	Flow cytometry
☒	☐	MRI-based neuroimaging

Clinical data

Policy information about clinical studies
All manuscripts should comply with the ICMJE guidelines for publication of clinical research and a completed CONSORT checklist must be included with all submissions.

Clinical trial registration: NCT03919877

Study protocol: https://clinicaltrials.gov/ct2/show/NCT03919877

Data collection: Data were collected at The Stanford Clinical Translational Research Unit (CTRU) and at home via CGM. Recruitment started in April 2019, and the last participant was recruited in May 2023.

Outcomes: The trial has four phases; the first is to identify metabolic sub-phenotype, and the last is to personalize the diet for each participant. The primary goal of the trial is to reduce the glucose level with the best diet for each metabolic sub-phenotype. In this study, we report the findings of the metabolic sub-phenotyping part.

Plants

Seed stocks: NA

Novel plant genotypes: NA

Authentication: NA
